# MicroRNA-centric measurement improves functional enrichment analysis of co-expressed and differentially expressed microRNA clusters

**DOI:** 10.1186/1471-2164-13-S7-S17

**Published:** 2012-12-07

**Authors:** Su Yeon Lee, Kyung-Ah Sohn, Ju Han Kim

**Affiliations:** 1 Seoul National University Biomedical Informatics (SNUBI) and Systems Biomedical Informatics Research Center, Div. of Biomedical Informatics, Seoul National University College of Medicine, Seoul 110799, Korea; 2 Institute of Endemic Diseases, Medical Research Center, Seoul National University, Seoul 110799, Korea

## Abstract

**Background:**

Functional annotations are available only for a very small fraction of microRNAs (miRNAs) and very few miRNA target genes are experimentally validated. Therefore, functional analysis of miRNA clusters has typically relied on computational target gene prediction followed by Gene Ontology and/or pathway analysis. These previous methods share the limitation that they do not consider the many-to-many-to-many tri-partite network topology between miRNAs, target genes, and functional annotations. Moreover, the highly false-positive nature of sequence-based target prediction algorithms causes propagation of annotation errors throughout the tri-partite network.

**Results:**

A new conceptual framework is proposed for functional analysis of miRNA clusters, which extends the conventional target gene-centric approaches to a more generalized tri-partite space. Under this framework, we construct miRNA-, target link-, and target gene-centric computational measures incorporating the whole tri-partite network topology. Each of these methods and all their possible combinations are evaluated on publicly available miRNA clusters and with a wide range of variations for miRNA-target gene relations. We find that the miRNA-centric measures outperform others in terms of the average specificity and functional homogeneity of the GO terms significantly enriched for each miRNA cluster.

**Conclusions:**

We propose novel miRNA-centric functional enrichment measures in a conceptual framework that connects the spaces of miRNAs, genes, and GO terms in a unified way. Our comprehensive evaluation result demonstrates that functional enrichment analysis of co-expressed and differentially expressed miRNA clusters can substantially benefit from the proposed miRNA-centric approaches.

## Background

MicroRNAs (miRNAs) are short single stranded, non-coding RNAs that regulate protein-coding mRNAs [1-4]. Mature miRNAs cause either target mRNA degradation or translational repression [4] by inducing cleavage or inhibiting translation in the 3'-untranslated regions (UTRs) of the target mRNA [2,3]. In spite of the continuous attempts to identify miRNAs and to elucidate their basic mechanisms of action, little is understood about their biological functions.

Because of the regulatory role of miRNAs [5] and lack of direct functional annotation to miRNAs, current functional enrichment methods for miRNAs rely instead on their target genes' functional annotations [6-8]. If the target genes of a specific miRNA are significantly enriched with a set of Gene Ontology (GO) terms, it is reasonable to infer that the miRNA is also involved in the same GO annotations. As only few experimentally validated targets are available, current methods of target gene's annotation-based inference of miRNA function rely on target prediction algorithms such as TargetScan [9,10] and Pictar [11].

Many studies on miRNAs have used this "predicted target-genes’ functional annotation-based" miRNA function prediction strategy. Gaidatzis *et al. *[12] applied a log-likelihood test for functional enrichment analysis for KEGG pathways. Gusev [13] used hypergeometric distributions for GO and pathway-based enrichment analysis. Xu and Wong [14] applied hypergeometric distribution test to detect significant over-representation of miRNA cluster targets in BioCarta pathways. Similar methods using GO, KEGG and BioCarta pathways were implemented in miRGator [15] and SigTerms [16], applying hypergeometric distributions to evaluate functional enrichment.

The target links from miRNAs to genes, however, show very uneven distributions. So do the links from genes to GO terms. One miRNA may regulate more than several hundreds of targets and one gene may be controlled by many miRNAs [17]. In contrast, the current methods that rely only on the predicted target genes' functional annotations are not powerful enough to capture such variability. For instance, if a certain miRNA targeting hundreds of genes is shared by different miRNA clusters, the clusters' functional annotations may become very similar even though they consist of very different miRNA members, just because they share the 'very bush' one. Another limitation of the current methods is that they treat all target genes equally. One should differently weight genes that are targeted by only one member from those that are targeted by all members of a miRNA cluster. In summary, the current functional enrichment methods for miRNA cluster have limitations of not considering the tri-partite network topologies from miRNAs to genes to functional annotations regarding multiplicity and cooperativity, containing more information than simple target gene counts.

**Figure 1:**
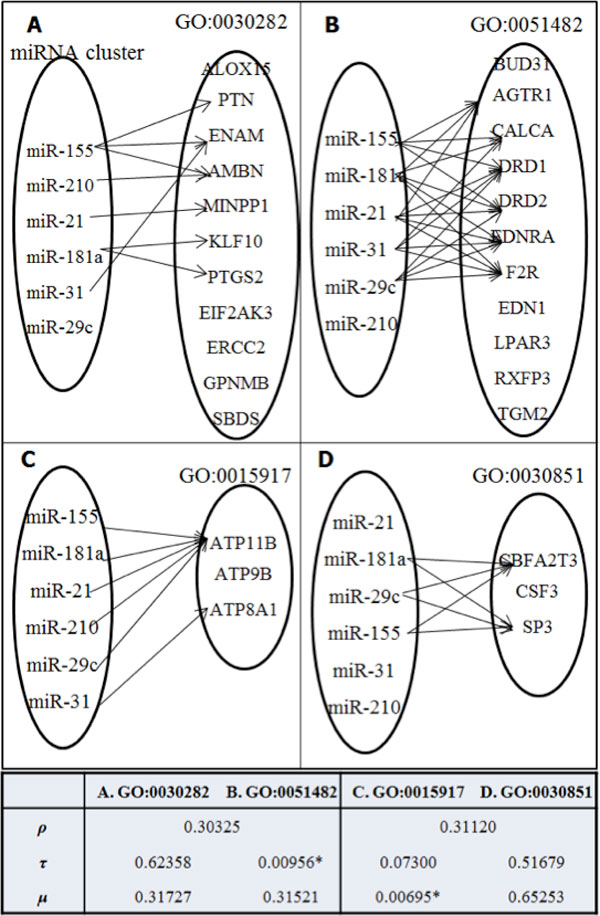
**Indiscernibility example**. Calculating target gene-centric (*ρ*) hypergeometric distribution cannot discern the completely different targeting topologies between (A) and (B) and between (C) and (D), resulting the same *p*-values (*p *= 0.30325 and 0.31120), respectively. The target link-centric (*τ p*-values can discriminate (A) and (B) (i.e., *p *= 0.62358 and 0.00956, respectively) and the miRNA-centric (*μ p*-values can discriminate (C) and (D) (i.e., *p *= 0.00695 and 0.65253, respectively). *p < 0.05, hypergeometric test.

For the purpose of illustration, Figure 1(A) and 1(B) exhibit example cases where the same numbers of miRNAs (*k *= 5) from equal-sized clusters (*k *= 6) are targeting the same numbers of target genes (*k *= 6) from equal number of genes (*k *= 11) that are annotated to a specific GO term, GO:0030282 and GO:0051482, respectively. The numbers of target links between Figure 1(A) and 1(B), however, are differently 8 and 22, respectively. Figure 1(C) and 1(D) exhibit cases where the numbers of miRNAs connected to a specific GO term, GO:0015917 and GO:0030851, are differently 6 and 3, respectively, while the numbers of links (*k *= 6) are the same. It is clearly demonstrated that the current approach only based on target gene counts is unable to discern the difference in these targeting relations.

The present study proposes a more generalized conceptual framework to develop and analyze new functional enrichment measures. According to the framework, the traditional "predicted target-genes' functional annotation-based" miRNA function prediction method is regarded as 'target gene-centric' denoted by *ρ *because it eventually considers only the fraction of the target genes among those that are annotated to a specific GO. Under the proposed framework, we derive 'target link-centric' (*τ*) and 'miRNA-centric' (*μ*) measures, considering the numbers of links and miRNAs linked to a specific GO term.

Figure 1 illustrates that while the traditional target gene-centric *ρ *measure cannot discern (A) and (B) (*p *= 0.30325) nor (C) and (D) (*p *= 0.31120), the newly proposed *τ *and *μ *measures successfully discern (A) and (B) (i.e., *p *= 0.62358 and *p *= 0.00956, respectively) and (C) and (D) (i.e., *p *= 0.00695 and *p *= 0.65253, respectively). It is clearly demonstrated that different measures calculated from different viewpoints significantly impact the result of functional enrichment analysis of miRNA clusters. We also propose a rank statistic for the purpose of systematic comparison in terms of the average specificity and functional homogeneity of the significantly enriched term for each GO category, Biological Process (BP), Molecular Functions (MF), and Cellular Components (CC). We show that the proposed miRNA-centric measures identify more specific and functionally homogenous sets of GO annotations for miRNA clusters.

## Methods

### Dataset: miRNA clusters

We used publicly available co-expressed and differentially expressed miRNA clusters for comparative evaluation of the proposed methods. For co-expressed miRNA clusters, we obtained the data created by Ruepp *et al. *[18] that show correlated expression patterns across several human diseases. The data can be downloaded from Ruepp *et al. *[18] (http://genomebiology.com/content/supplementary/gb-2010-11-1-r6-s2.xls). Forty three among the 47 clusters having at least one target gene were used in this study. Differentially expressed miRNA sets consisting of up- or down-regulated genes in six solid tumors were also downloaded [19]. MiRNAs down-regulated in colon cancer had no target gene and hence were excluded in the present study. Supplement Tables S1 and S2 in 'Additional file 1' list the 54 (= 43 + (2 × 6) - 1) miRNA clusters from the two studies with the associated information.

### Creating variations of miRNA-mRNA target pairs for comprehensive evaluation

Another input of our analysis is the target gene list of each miRNA that will guide the functional enrichment test based on the gene annotations. Considering that only a few experimentally validated miRNA targets are available, we use miRNA-mRNA target pairs obtained from computational target prediction methods. Prediction algorithms generate a relatively high level of false positives [20] and the degree of overlap between predicted targets from different methods is often poor or null [21]. Given the lack of 'gold standard' for miRNA and target gene pairs, we consider a wide range of variations in miRNA-gene pair relations for comprehensive evaluation. We used miRecords [22] and miRGen [23], which are integrated resources of miRNA-target interactions from 11 established target prediction algorithms and from four most widely used target prediction programs, respectively. We created 21 variations for predicted target pairs by considering the number of positive voters from the included algorithms by miRecords (Table 1, upper panel) and six variations by applying the four programs of miRGen (Table 1, lower panel). Because most of the evaluation results from these variations were largely comparable, the most representative variation #6 in Table 1 was used to describe the overall study results in the following sections. Variation #6 was created by applying the 11 algorithms provided by miRecords, wining more than three positive voters and resulting in 1,569,741 target links from 553 miRNAs to 17,636 genes. As the number of required positive voters is increasing, the numbers of miRNAs, links and genes are decreasing as can be seen in Table 1.

**Table 1:** Variation for predicted miRNA-gene target pairs

Index	No. of algorithms showing positive voting	Numbers of		
	
		miRNAs	Target links	Genes
miRecords (Xiao *et al*., 2009)				

#1	3 algorithms	553	1,234,390	17,602
#2	4	535	272,505	15,278
#3	5	407	53,041	9,747
#4	6	159	9,691	2,783
#5	7	29	68	66
**#6**	**3 ~ 11**	**553**	**1,569,741**	**17,636**
#7	4 ~ 11	535	335,351	15,422
#8	5 ~ 11	408	62,846	9,851
#9	6 ~ 11	159	9,805	2,816
#10	7 ~ 11	40	114	104
#11	3 ~ 11 including DIANA-microT	0	0	0
#12	3 ~ 11 including Microinspector	56	184	160
#13	3 ~ 11 including miRanda	552	1,416,379	17,584
#14	3 ~ 11 including mirtarget2	530	184,544	13,841
#15	3 ~ 11 including miTarget	0	0	0
#16	3 ~ 11 including NBmiRTar	42	201	172
#17	3 ~ 11 including PicTar	163	64,658	6,515
#18	3 ~ 11 including pita	551	1,559,586	16,676
#19	3 ~ 11 including rna22	54	232	197
#20	3 ~ 11 including rnahybrid	552	1,548,423	17,630
#21	3 ~ 11 including TargetScan	412	343,190	16,127

miRGen [23]				

#22	DIANA-microT	175	1,816	1,206
#23	miRanda (microrna.org)	469	430,878	16,699
#24	miRanda (miRBase)	156	38,821	5,444
#25	PicTar (4-way)	177	68,100	6,391
#26	PicTar (5-way)	128	22,028	2,433
#27	TargetScanS	237	75,044	7,546

### Target gene-, target relation-, and miRNA-centric calculations of hypergeometric distributions

**Figure 2:**
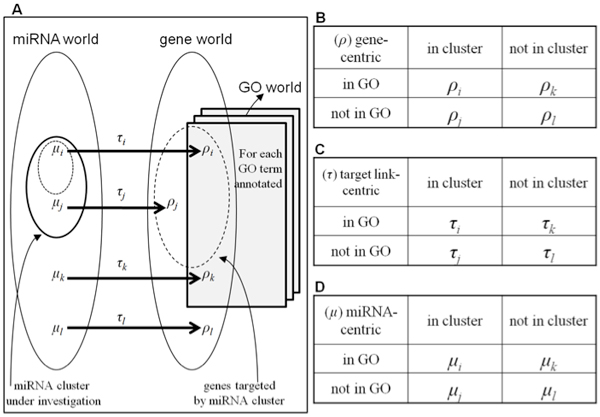
**Framework for developing three types of miRNA functional enrichment measures**. A conceptual framework is constructed to consider the tri-partite network topology. (A) A miRNA cluster under investigation contains the members, *μ_i _*and *μ_j_*, targeting genes that are associated (*ρ_i_*) and not associated (*ρ_j_*) with a specific GO term of interest through *τ_i _*and *τ_j_*, respectively. Non-member miRNAs may be associated (*μ_k_*→*ρ_k_*) or not (*μ_l_*→*ρ_l_*) with the GO term through *τ_k _*and *τ_l_*. Counts for (D) miRNA-centric (*μ*) and (C) target link-centric (*τ*) as well as (B) target gene-centric (*ρ*) are listed by two-by-two contingency tables. The closed and broken circles in the miRNA world depict the miRNA cluster under investigation and the subset miRNAs targeting the genes that are associated with a specific GO term of interest.

Now we describe the details of the proposed measures in a proposed conceptual framework. Suppose we want to test the functional enrichment of a miRNA cluster with respect to a specific GO term (or annotation). In most previous approaches, one first constructs a corresponding target gene cluster consisting of all the genes targeted by at least one member in the miRNA cluster. Then the numbers of target genes annotated (*ρ_i_*) and not annotated (*ρ_j_*) by the GO term are used in the two by two contingency table along with the numbers of genes not in the target cluster and are either annotated (*ρ_k_*) or not annotated (*ρ_l_*) with the term, as shown in Figure 2(B). Functional enrichment is tested from this contingency table using a hypergeometric distribution. These traditional target gene-centric (*ρ*) methods are limited in that they consider only the fraction of target genes connected to a specific annotation for each annotation [12-14], as already illustrated in Figure 1. To this rather confusing problem, the diagram and contingency tables in Figure 2 provide a conceptual framework to understand and correctly design new functional enrichment measures. The diagram of miRNA, gene and annotation worlds in Figure 2(A) depicts the tri-partite network topology between the three worlds such that one can drive the quartet numbers to create contingency tables for miRNA-centric (*τ*) and target link-centric (*μ*) as well as for the target gene-centric (*ρ*) measures (Figure 2(B)~(D)).

Under this conceptual framework in Figure 2, subscripts *i *and *k *represent positive and subscripts *j *and *l *negative connections to the GO term. Subscripts *i *and *j *represents connections from inside of and *k *and *l *from outside of the targeting miRNA or target gene clusters. The traditional *ρ_i _*and *ρ_j_*, for example, correspond to the sets of target genes that are annotated (*ρ_i_*) and not annotated (*ρ_j_*) to a specific GO term. *ρ_k _*and *ρ_l _*denote non-targeted genes that are annotated (*ρ_k_*) and not annotated (*ρ_l_*) to the GO term. We can develop a miRNA-centric measure in the conceptualized three framework in a consistent way. We define *μ_i _*and *μ_j _*as the miRNAs in the cluster whose target genes are annotated (*μ_i_*) and not annotated (*μ_j_*) to the GO term. As in the case of a gene-centric measure, *μ_k _*and *μ_l _*correspond to miRNAs outside of the cluster whose target genes are annotated (*μ_k_*) and not annotated (*μ_k_*) to the GO term. Similarly, for a target link-centric measure, we define *τ_i _*and *τ_j _*as the target links connecting members of the miRNA cluster in *μ_i _*and in *μ_j_*, respectively, to genes that are connected (*ρ_i_*) and not connected (*ρ_j_*) to a specific GO term. Remaining miRNAs outside the cluster, *μ_k _*and *μ_l_*, target genes through *τ_k _*and *τ_l _*that are headed to genes that are connected (*ρ_k_*) and not connected (*ρ_l_*) to the GO term.

To formally define the three measures, let *ρ, τ*, and *μ *be the random variables that represent the number of target genes, target links, miRNAs, respectively, which are linked to a specific GO term as explained above. The following three equations, (1), (2), and (3), describe the hypergeometric distributions of *ρ, τ*, and *μ*, respectively.

(1) $$probability\left(\rho =\rho_{i}\right)=\frac{\left(\begin{array}{c} \rho_{i}+\rho_{k}\\ \rho_{i} \end{array}\right)\left(\begin{array}{c} \rho_{j}+\rho_{l}\\ \rho_{j} \end{array}\right)}{\left(\begin{array}{c} \rho_{i}+\rho_{j}+\rho_{k}+\rho_{l}\\ \rho_{i}+\rho_{j} \end{array}\right)}$$

(2) $$probability\left(\tau =\tau_{i}\right)=\frac{\left(\begin{array}{c} \tau_{i}+\tau_{k}\\ \tau_{i} \end{array}\right)\left(\begin{array}{c} \tau_{j}+\tau_{l}\\ \tau_{j} \end{array}\right)}{\left(\begin{array}{c} \tau_{i}+\tau_{j}+\tau_{k}+\tau_{l}\\ \tau_{i}+\tau_{j} \end{array}\right)}$$

(3) $$probability\left(\mu =\mu_{i}\right)=\frac{\left(\begin{array}{c} \mu_{i}+\mu_{k}\\ \mu_{i} \end{array}\right)\left(\begin{array}{c} \mu_{j}+\mu_{l}\\ \mu_{j} \end{array}\right)}{\left(\begin{array}{c} \mu_{i}+\mu_{j}+\mu_{k}+\mu_{l}\\ \mu_{i}+\mu_{j} \end{array}\right)}$$

Note that for notational convenience, we now used *ρ_a_, τ_a_, μ_a _*for *a *∈ {*i, j, k, l*}, instead of |*ρ_a_*|, etc., to represent the number of members in the corresponding set by abuse of notation. The *p*-value for the enrichment test from hypergeometric distribution of the random variable *ρ *is calculated from the cumulative probability of observing at least *ρ_i _*out of *ρ_i _*+ *ρ_j _*times. Accordingly, the *p*-value from each of the three measures can be defined as follows;

$$p-value_{\rho }=probability\left(\rho \geq \rho_{i}\right)$$

$$p-value_{\tau }=probability\left(\tau \geq \tau_{i}\right)$$

$$p-value_{\mu }=probability\left(\mu \geq \mu_{i}\right)$$

These probabilities are computed using the phyper and dhyper functions in R 'stats' package.

### Combining P-values

**Figure 3:**
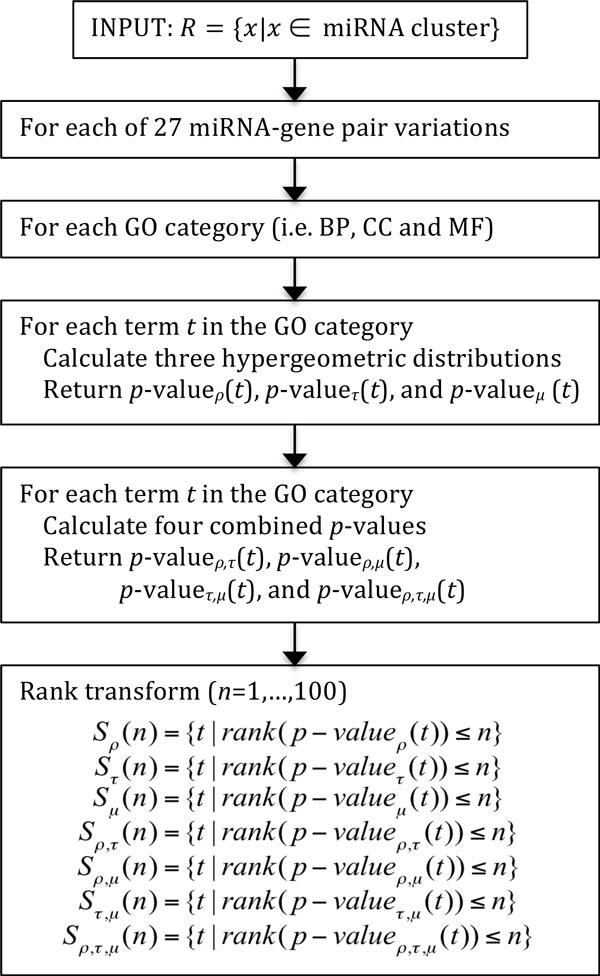
**Steps for combining three types of *p*-values**. For a selected GO category and a miRNA-gene target-pair variation, for each GO term, three *p*-values are computed for *ρ, τ*, and *μ*, and then rank normalized. *S_ρ_*(*n*) denotes the set of GO terms whose *p*-values' ranks in the *ρ *hypergeometric distribution are less than or equal to *n*. By applying set operations, four combinations of *S_ρ_*(*n*), *S_τ_*(*n*), *S_μ_*(*n*) are created for further evaluation.

For the purpose of comprehensive evaluation, we create all possible combinations of the three measures and tested each of those at all GO categories and using different miRNA-target gene pair sets. Figure 3 illustrates steps of combining the three types of hypergeometric distributions for *ρ, τ *and *μ*. For each of the 54 miRNA clusters, of the 27 variations for miRNA-target gene pairs, of the three GO categories, and of annotations (or GO terms), three *p*-values, *p_ρ_, p_τ _*and *p_μ_*, are first computed. Then, we generate 4 combined *p*-values by using Fisher's combined *p*-value method [24].

$$\begin{array}{l} \;\;\;p_{\rho ,\tau }:\text{ combined p-value of }p_{\rho }\text{ and }p_{\tau }\\ \;\;\;p_{\rho ,\mu }:\text{ combined p-value of }p_{\rho }\text{ and }p_{\mu }\\ \;\;\;p_{\tau ,\mu }:\text{ combined p-value of }p_{\tau }\text{ and }p_{\mu }\\ p_{\rho ,\tau ,\mu }:\text{ combined p-value of }p_{\rho },p_{\tau }\text{ and }p_{\mu } \end{array}$$

We briefly describe how Fisher's combined *p*-value method can be applied to our proposed measures. Under the null hypothesis of no significant enrichment, the individual *p*-value for the random variable *ρ, τ*, or *μ *follows the uniform distribution on 0[1]. Then the distribution of

Y=−ln(p-value)

is chi-squared with one degree of freedom. We have three *p*-values from *ρ, τ*, and *μ *hypergeometric distributions,

$$p_{\rho ,}p_{\tau }\text{ and }p_{\mu ,}$$

and thus we define

$$Y_{\rho }=-ln\left(p_{\rho }\right),Y_{\tau }=-ln\left(p_{\tau }\right),\text{ and }Y_{\mu }=-ln\left(p_{\mu }\right)$$

Each of the random variables *Y_ρ_, Y_τ_*, and *Y_μ _*is under the chi-squared distribution with one degree of freedom. The final four sums of *W *are then defined as follows*:*

$$\begin{array}{c} W_{1}=\;Y_{\rho }+Y_{\tau }\\ W_{2}=\;Y_{\rho }+Y_{\mu }\\ W_{3}=\;Y_{\tau }+Y_{\mu }\\ W_{4}=\;Y_{\rho }+Y_{\tau }+Y_{\mu } \end{array}$$

The random variables *W*_1_, ..., *W*_4 _follow chi-squared distribution with degrees of freedom 2, 2, 2, and 3, respectively. These random variables are used to produce the combined 'overall' *p*-values. To calculate these *p*-values, we applied fisherSum function in R 'MADAM' package [25].

The underlying distribution of p-values from each method can be different due to the different characteristics of the measure. To take into account this heterogeneity in the distribution of *p*-values, we rank-normalized *p*-values for each GO category as shown in the last step of Figure 3. Specifically, we construct the set *S_θ_*(*n*) of top *n *significant GO terms having the smallest *p*-values for each measure *θ *∈ {*ρ, τ, μ*}. Four additional sets of *S_ρ,τ_*(*n*), *S_ρ,μ_*(*n*), *S_τ,μ_*(*n*), and *S_ρ,τ,μ_*(*n*) for the combined measures are also created and used for further evaluation.

### Evaluation measures

Average specificities and functional homogeneity index (or semantic similarity density) of the rank normalized term sets *S_θ_*(*n*) for each measure *θ *∈{*ρ, τ, μ*,(*ρ, τ*), (*ρ, μ*), (*τ, μ*), (*ρ, τ, μ*)} are computed for performance comparison. This is based on the general assumption that for a specific set of GO terms identified by each measure, the more functionally homogenous the set is, the more reliable the measure is. In addition, higher specificities are more desirable because it is more informative to have more specific terms than more general terms in the functional analysis of clusters.

Many studies have shown that Information Content (IC) can quantify the specificity of a cluster [26,27]. IC measure is based on the fact that less frequently used terms are more specific. The IC of a GO term *t *is defined as follows:

(4) $$IC\left(t\right)=-\text{log}\left(\frac{freq\left(t\right)}{freq\left(root\right)}\right)$$

where *root *represents the root term for each GO category. *freq*(*t*) is defined as follows;

(5) $$freq\left(t\right)=n\left(annotate\left(t\right)\right)+\underset{c\in \text{children}\left(t\right)}{\sum }n\left(annotate\left(c\right)\right)$$

where *children*(*t*) returns the list of child terms of term *t*. Thus *t *becomes a parent term of all members of *children t*), either directly or indirectly. The functions *annotate*(*t*) and *n*(*G*) return the list of genes that are annotated to GO term *t *and the number of the genes in the gene list *G*, respectively. We use the average IC value of the given term set as a performance measure to compare the specificity.

For functional homogeneity index (or semantic similarity density), we choose a widely used Resnik's measure of semantic similarity [28]. The semantic similarity between two terms is defined as the IC of the lowest common ancestor (LCA) of the two terms and hence is obtained by:

(6) $$S_{Resnik}\left(t_{A},t_{B}\right)=IC\left(LCA\left(t_{A},t_{B}\right)\right)$$

As an evaluation measure, the average of all pairwise term-to-term Resnik's similarities was applied for *S_θ_*(*n*) for each measure *θ *∈ {*ρ, τ, μ, (ρ, τ*), (*ρ, μ*), (*τ, μ*), (*ρ, τ, μ*)} and defined as semantic similarity density of the set.

GO terms and associated gene sets were downloaded from http://www.geneontology.org/gene-associations/gene_association.goa_human.gz. We excluded GO associations having ND (No biological data) or NR (Not Recorded) evidence codes.

## Results

### Average specificity and functional homogeneity index distributions

**Figure 4:**
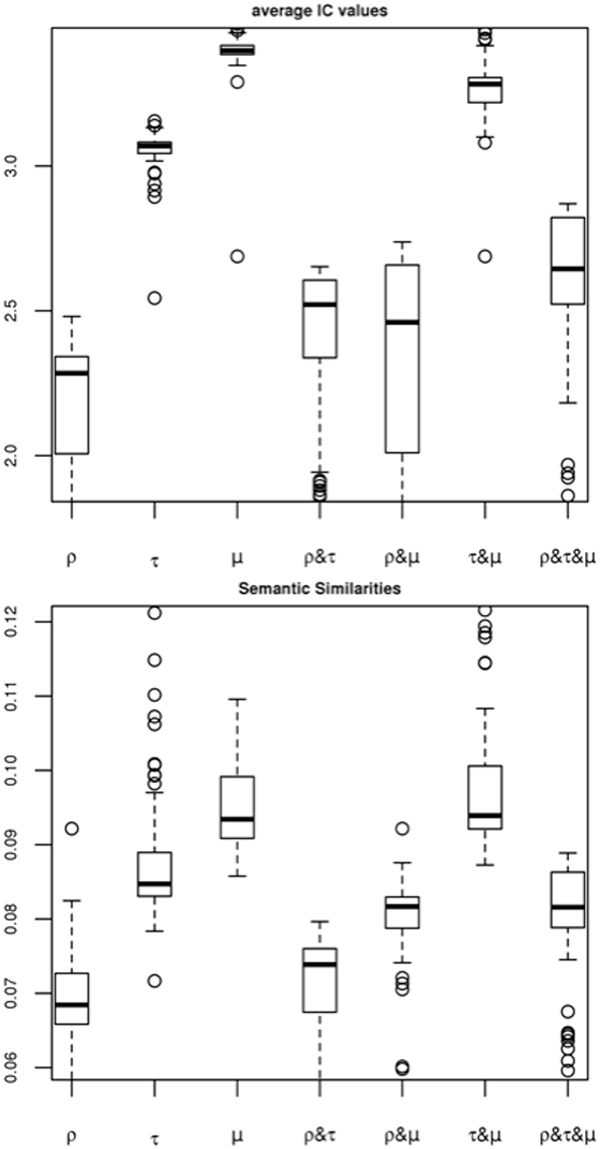
**Evaluation of functional enrichment measures and their combinations**. Distributions of (A) functional homogeneity index (or average IC value) and (B) semantic similarity (or average all pair-wise Resnik's similarity) are exhibited for significantly enriched GO BP terms in the 'breast/up-regulated miRNA cluster' from Volinia *et al. *[19](see index 1 in Supplement Table S2) by applying target variation #6 in Table 1. MicroRNA-centric measure (*μ*) outperforms the traditional target gene-centric measure (*ρ*) and others.

Figure 4 shows the distributions of average IC values and functional homogeneity index for GO BP terms with *p*-values in top *n *= 100 ranks in the 'breast/up-regulated miRNA cluster' from Volinia *et al. *[19] (Supplementary Table S2 in 'Additional file 1'). Most of the highest average IC and functional homogeneity values were obtained by miRNA-centric *μ *measures throughout the evaluations (see Supplement Fig. S1 series in 'Additional file 1') including the specific example shown in Figure 4. Because of the small numbers of miRNA members and target genes, target variations #5, #10, #11, #15, and #16 in Table 1 had no significant GO terms. Evaluation showed that miRNA-centric *μ *measure exhibited the best specificity and homogeneity except only for the target variations #12, #19 and #22. The very small numbers of miRNAs (i.e., *m *= 56, 54, 175, respectively) and target genes (i.e., *m *= 160, 197, 1206, respectively) from the very strict thresholds may explain the results. These findings are also consistent throughout the evaluation study regardless of different GO categories.

### Performance comparison with a varying parameter setting

**Figure 5:**
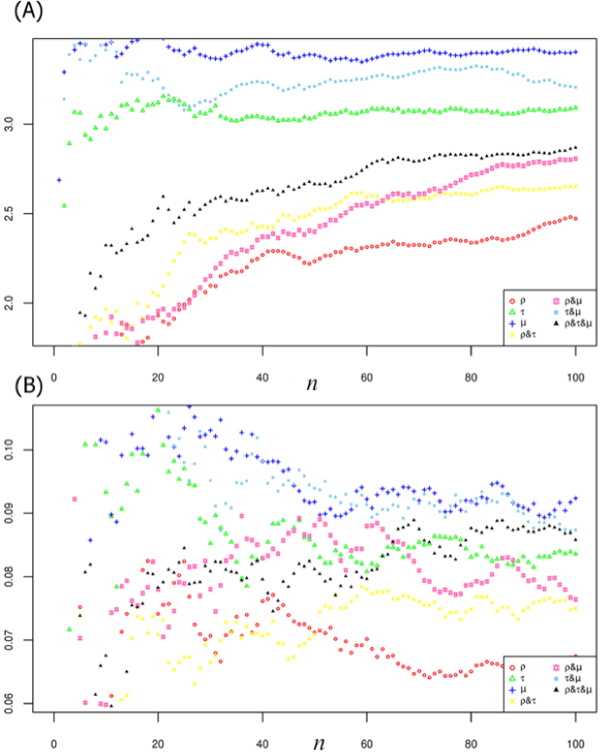
**Evaluation of functional homogeneity and semantic similarity densities across different thresholds**. Average (A) information content and (B) all pair-wise semantic similarity values are plotted with increasing numbers of rank normalized GO terms *n *(see Fig. 3) for "breast/up-regulated miRNA cluster" from Volinia *et al. *[19] (index 1 in Supplementary Table S1 in 'Additional file 1') by applying target variation #6 in Table 1, GO BP category. Measures containing miRNA-centric *μ *(in blue) like (*ρ, μ*) (in pink) and (*τ, μ*) (in sky blue) consistently outperform traditional gene-centric *ρ *(in red) measures at all levels.

**Figure 6:**
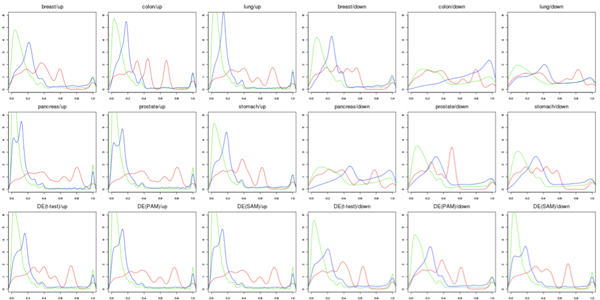
**Distribution of *p*-values for all GO BP terms**. Distribution of *p*-values for all GO BP terms demonstrates that miRNA-centric *μ *(in green) shows overall better discriminant power than target link-centric *τ *(in blue) and traditional gene-centric *ρ *(in red) methods for datasets from Volinia *et al. *[19].

Figure 5(A) and 5(B) shows the distributions of the average IC values and functional homogeneity values with increasing numbers of rank normalized GO terms *n *(see Figure 3), as an example for "breast/up-regulated miRNA cluster" from Volinia *et al. *[19] (index 1 in Supplementary Table S1 in 'Additional file 1') by applying target variation #6 in Table 1, GO BP category. Measures containing miRNA-centric *μ *(in blue cross) like (*ρ, μ*) and (*τ, μ*) consistently outperformed traditional gene-centric *ρ *(in red circle) at all threshold levels of *n*. Figure 6 demonstrates the distribution of *p*-values for all GO BP terms annotated to the miRNA clusters from the dataset of Volinia *et al. *[19]. Although the interpretation about the p-value distribution is generally tricky and needs to be done carefully, it seems that the *p*-value distribution for miRNA-centric *μ *(in green) shows overall better discriminant power than target link-centric *τ *(in blue) and traditional gene-centric *ρ *(in red) methods.

### Examples showing complementary properties

Examples of GO terms determined to be statistically significant by miRNA-centric *μ *but not by traditional gene-centric *ρ *method are listed in the upper part of Table 2. Gusev [13] correctly pointed out that it was common for top ranked GO terms to be targeted by every member of the corresponding miRNA cluster. Those that are targeted by all six miRNA members (i.e., *μ_i _*= 6) shown in the upper part of Table 2, however, are not statistically significant (*p *> 0.05) and show poor ranks (>290) by *ρ *method. But *μ *method shows statistical significances (*p *< 0.05) with high ranks (<35) (Table 2). In contrast, those that are targeted by all six miRNA members shown in the middle part of Table 2 show very strong statistical significance (*p *< 0.001) by *ρ *method. The very low *μ_k _*to *μ_l _*ratios (i.e., about 50:1) in the middle part compared to those in the upper part (i.e., about 1:1) of Table 2 clearly explain the poor *p*-values and ranks (>2500) by *μ *method. Therefore, Gusev's correct intuition can further be formally analyzed by introducing miRNA-centric *μ *method. It is demonstrated that our new measure considering *μ *complements some drawbacks of the traditional gene-centric *ρ *measure.

**Table 2:** Comparison of miRNA-centric *μ *and gene-centric *ρ *measures^a^

GO terms	*ρ_i_*	*ρ_j_*	*ρ_k_*	*ρ_l_*	*p-*value*_ρ _*(rank)
	***μ_i_***	***μ_j_***	***μ_k_***	***μ_l_***	***p*-value*_μ _*(rank)**
GO:0033137, *negative regulation *	3	7485	0	10148	0.0765 (299)
*of peptidyl-serine phosphorylation*	6	0	282	265	0.0195 (14)
GO:0006474, *N-terminal protein *	3	7485	0	10148	0.0765 (299)
*amino acid acetylation*	6	0	320	227	0.0412 (28)
GO:0008054, *cyclin catabolic *	2	7486	2	10146	0.5668 (1842)
*process*	6	0	322	225	0.0427 (32)

GO:0031047, gene silencing by RNA	19	7460	6	10142	0.0006 (25)
	6	0	537	10	0.8958 (2566)
GO:0030335, positive regulation of cell migration	34	7454	16	10132	2.31e-04 (18)
	6	0	545	2	0.9783 (2879)
GO:0045944, positive regulation of	150	7338	112	10036	8.62e-07 (4)
transcription from RNA polymerase II promoter	6	0	546	1	0.9891 (2923)

GO:0006956, *complement *	1	7487	0	10148	0.4246 (1251)
*activation*	2	4	3	544	0.0010 (1)
GO:0060022, *hard palate *	1	7487	0	10148	0.4246 (1251)
*development*	4	2	85	462	0.0073 (3)
GO:0060123, *regulation of growth *	1	7487	0	10148	0.4246 (1251)
*hormone secretion*	4	2	87	460	0.0079 (4)
GO:0032926, *negative regulation *	3	7485	0	10148	0.07652 (286)
*of activin receptor signaling pathway*	5	1	226	321	0.0437 (35)
GO:0015936, *coenzyme A *	1	7487	1	10147	0.6689 (2086)
*metabolic process*	5	1	160	387	0.0102 (5)

^a ^The 'breast/up-regulated' miRNA cluster data from Volinia *et al. *(2006) using the target variation #6 (see Table 1) was used.

The GO terms in the lower part of Table 2 are annotated only to two to five among six mRNA members such that they are far from statistical significance by *ρ *calculations. The *p*-values by *μ *method, however, are even more statistically significant. *Complement activation *(GO:0006956) in GO BP category was rejected by the traditional *ρ *method (*p *= 0.42) but accepted by miRNA-centric *μ *method (*p *> 0.001) with ranks of 1251 and 1, respectively. *Complement activation *indeed has long been well recognized in breast cancer [29,30]. At least four well-known breast cancer genes including SMAD2, SMAD4, TGFB3 and TGFBR3 are involved in *palate development*. There are many studies reporting that *regulation of growth hormone secretion *(GO:0060123) is indeed associated with breast cancer [31-33]. For the GO term, *negative regulation of activin receptor signaling pathway *(GO:0032926), many studies reported that facilitating activin signaling either by Cripto silencing or FLRG silencing inhibits human breast cancer cell growth [34,35]. Numerous studies have reported that acetyl-CoA carboxylase (ACCα) and fatty acid synthase (FAS), key limiting fatty acid synthesis enzymes involved in *coenzyme A metabolic process *(GO:0015936), are highly expressed in human breast cancer cell lines and breast carcinomas [36-40]. Moreover, pantothenate kinase 3 (PANK3) and Coenzyme A synthase (COASY) are known breast cancer genes.

## Discussion

We proposed miRNA-centric *μ *and target link-centric *τ *measures that improve functional enrichment analysis of differentially expressed or co-expressed miRNA clusters. We performed comprehensive evaluations of different methods on various settings. It is demonstrated that these new measures complement the conventional target gene-centric *ρ *measure and miRNA-centric *μ *method was among the most powerful and reliable.

MicroRNA's intrinsic properties of multiplicity and cooperativity [17] may be correctly modeled by combined hypergeometric distributions. Average IC value for the *μ *category was consistently the highest among different conditions and measures. It is suggested that the number of miRNAs and their relations associated with a specific GO term of interest is as much important as the number of target mRNAs associated with the GO term. Therefore, applying *ρ, τ*, and *μ *hypergeometric distributions for functional annotation of miRNAs are mutually complementary.

The proposed method is based on computationally predicted rather than experimentally validated target relations. Computational prediction has limitations given high level of false positives and negatives. Especially, it is difficult to obtain predicted targets for minor forms of miRNA such as star, -3p, -5p or other recently identified forms of miRNAs. All current computational enrichment analysis methods that use predicted target relations suffer from the same drawback. Combining the proposed three methods may complement with each other in finding and evaluating the correct miRNA-mRNA target relations, and improving functional annotations and enrichment analysis.

## Competing interests

The authors declare that they have no competing interests.

## Authors' contributions

SL and JK conceived and designed the study. SL performed the experiments. KS and JK helped to refine the analysis and the interpretation of results. SL, KS, and JK wrote the manuscript.

## Supplementary Material

Additional file 1 **Supplementary Figures and Tables**. This file contains additional figures and tables mentioned in the main text. Click here for file
