# Supplementary material for: MicroRNA-centric measurement improves functional enrichment analysis of co-expressed and differentially expressed microRNA clusters
Source: BMC Genomics. 2012 Dec 7;13(Suppl 7):S17. doi: 10.1186/1471-2164-13-S7-S17 (PMC3521213; doi:10.1186/1471-2164-13-S7-S17)

**Additional File 1:**

**Supplementary Tables**

**Table S1.** miRNA clusters and the number of miRNAs that have target mRNAs were provided from Ruepp *et al*.

| index | miRNA cluster | # of miRNA members in this cluster | # of miRNAs which have at least one target mRNA |
| --- | --- | --- | --- |
| 1 | hsa-mir-134,hsa-mir-154,hsa-mir-376c,hsa-mir-369,hsa-mir-376a-1,hsa-mir-377,hsa-mir-381,hsa-mir-382,hsa-mir-453,hsa-mir-409,hsa-mir-412,hsa-mir-410,hsa-mir-376b,hsa-mir-485,hsa-mir-487a,hsa-mir-496,hsa-mir-539,hsa-mir-544,hsa-mir-376a-2,hsa-mir-487b,hsa-mir-654,hsa-mir-655,hsa-mir-656,hsa-mir-668,hsa-mir-1185-2,hsa-mir-1185-1,hsa-mir-300,hsa-mir-541,hsa-mir-889 | 29 | 20 |
| 2 | hsa-mir-512-1,hsa-mir-512-2,hsa-mir-498,hsa-mir-520e,hsa-mir-515-1,hsa-mir-519e,hsa-mir-520f,hsa-mir-515-2,hsa-mir-519c,hsa-mir-520a,hsa-mir-526b,hsa-mir-519b,hsa-mir-525,hsa-mir-523,hsa-mir-518f,hsa-mir-520b,hsa-mir-518b,hsa-mir-526a-1,hsa-mir-520c,hsa-mir-518c,hsa-mir-524,hsa-mir-517a,hsa-mir-519d,hsa-mir-521-2,hsa-mir-520d,hsa-mir-517b,hsa-mir-520g,hsa-mir-516b-2,hsa-mir-526a-2,hsa-mir-518e,hsa-mir-518a-1,hsa-mir-518d,hsa-mir-516b-1,hsa-mir-518a-2,hsa-mir-517c,hsa-mir-520h,hsa-mir-1323,hsa-mir-1283-1 | 38 | 17 |
| 3 | hsa-mir-16-2,hsa-mir-15b | 2 | 1 |
| 4 | hsa-mir-105-1,hsa-mir-105-2,hsa-mir-767 | 3 | 0 |
| 5 | hsa-mir-127,hsa-mir-136,hsa-mir-431,hsa-mir-433,hsa-mir-432 | 5 | 4 |
| 6 | hsa-mir-299,hsa-mir-379,hsa-mir-380,hsa-mir-323,hsa-mir-329-1,hsa-mir-329-2,hsa-mir-494,hsa-mir-495,hsa-mir-411,hsa-mir-758,hsa-mir-543,hsa-mir-1197 | 12 | 7 |
| 7 | hsa-mir-521-1,hsa-mir-522,hsa-mir-519a-1,hsa-mir-527,hsa-mir-516a-1,hsa-mir-516a-2,hsa-mir-519a-2,hsa-mir-1283-2 | 8 | 2 |
| 8 | hsa-mir-34b,hsa-mir-34c | 2 | 1 |
| 9 | hsa-mir-144,hsa-mir-451 | 2 | 2 |
| 10 | hsa-mir-224,hsa-mir-452 | 2 | 2 |
| 11 | hsa-mir-421,hsa-mir-374b | 2 | 2 |
| 12 | hsa-mir-449a,hsa-mir-449b | 2 | 2 |
| 13 | hsa-mir-296,hsa-mir-298 | 2 | 1 |
| 14 | hsa-mir-424,hsa-mir-450a-1,hsa-mir-450a-2,hsa-mir-503,hsa-mir-542,hsa-mir-450b | 6 | 2 |
| 15 | hsa-mir-215,hsa-mir-194-1 | 2 | 1 |
| 16 | hsa-mir-221,hsa-mir-222 | 2 | 2 |
| 17 | hsa-mir-141,hsa-mir-200c | 2 | 2 |
| 18 | hsa-let-7c,hsa-mir-99a | 2 | 2 |
| 19 | hsa-mir-195,hsa-mir-497 | 2 | 2 |
| 20 | hsa-mir-143,hsa-mir-145 | 2 | 2 |
| 21 | hsa-mir-23a,hsa-mir-24-2,hsa-mir-27a | 3 | 2 |
| 22 | hsa-mir-181b-1,hsa-mir-181a-1 | 2 | 0 |
| 23 | hsa-let-7a-3,hsa-let-7b | 2 | 1 |
| 24 | hsa-mir-25,hsa-mir-93,hsa-mir-106b | 3 | 3 |
| 25 | hsa-mir-24-1,hsa-mir-23b,hsa-mir-27b | 3 | 2 |
| 26 | hsa-mir-181c,hsa-mir-181d | 2 | 2 |
| 27 | hsa-mir-191,hsa-mir-425 | 2 | 2 |
| 28 | hsa-mir-30c-1,hsa-mir-30e | 2 | 1 |
| 29 | hsa-let-7a-1,hsa-let-7d,hsa-let-7f-1 | 3 | 1 |
| 30 | hsa-mir-15a,hsa-mir-16-1 | 2 | 1 |
| 31 | hsa-mir-19b-2,hsa-mir-92a-2,hsa-mir-106a,hsa-mir-363,hsa-mir-18b,hsa-mir-20b | 6 | 4 |
| 32 | hsa-mir-302a,hsa-mir-302b,hsa-mir-302c,hsa-mir-302d,hsa-mir-367 | 5 | 5 |
| 33 | hsa-mir-29a,hsa-mir-29b-1 | 2 | 1 |
| 34 | hsa-mir-212,hsa-mir-132 | 2 | 2 |
| 35 | hsa-mir-29b-2,hsa-mir-29c | 2 | 1 |
| 36 | hsa-mir-188,hsa-mir-362,hsa-mir-500,hsa-mir-501,hsa-mir-502,hsa-mir-532,hsa-mir-660 | 7 | 2 |
| 37 | hsa-let-7e,hsa-mir-125a,hsa-mir-99b | 3 | 2 |
| 38 | hsa-mir-1-2,hsa-mir-133a-1 | 2 | 0 |
| 39 | hsa-mir-200b,hsa-mir-200a,hsa-mir-429 | 3 | 3 |
| 40 | hsa-mir-192,hsa-mir-194-2 | 2 | 1 |
| 41 | hsa-mir-17,hsa-mir-18a,hsa-mir-19a,hsa-mir-19b-1,hsa-mir-20a,hsa-mir-92a-1 | 6 | 4 |
| 42 | hsa-mir-96,hsa-mir-182,hsa-mir-183 | 3 | 3 |
| 43 | hsa-mir-30d,hsa-mir-30b | 2 | 2 |
| 44 | hsa-mir-181a-2,hsa-mir-181b-2 | 2 | 0 |
| 45 | hsa-mir-206,hsa-mir-133b | 2 | 2 |
| 46 | hsa-mir-371,hsa-mir-372,hsa-mir-373 | 3 | 2 |
| 47 | hsa-let-7f-2,hsa-mir-98 | 2 | 1 |

**Table S2.** miRNA clusters and the number of miRNAs that have target mRNAs were provided from Volinia *et al*.

| index | cluster description from Volinia *et al.* | # of miRNA members in this cluster | # of miRNAs which have at least one target mRNA |
| --- | --- | --- | --- |
| 1 | breast/up | 15 | 6 |
| 2 | breast/down | 12 | 7 |
| 3 | colon/up | 22 | 13 |
| 4 | colon/down | 1 | 0 |
| 5 | lung/up | 35 | 16 |
| 6 | lung/down | 3 | 1 |
| 7 | pancreas/up | 55 | 29 |
| 8 | pancreas/down | 2 | 1 |
| 9 | prostates/up | 39 | 21 |
| 10 | prostates/down | 6 | 1 |
| 11 | stomach/up | 22 | 13 |
| 12 | stomach/down | 6 | 3 |

**Supplementary Figures**

**Fig. S1.** Distributions of average IC values of GO annotations for miRNA clusters (listed in Supplementary Tables S1 and S2). Horizontal axis denotes three hypergeometric distributions. Vertical axis denotes average IC value for GO annotations to miRNA cluster. (blue text represents miRNA clusters in Table S1 and S2 ; total 54 clusters)

Fig S1 file name is composed by miRNA-mRNA target variation in Table 1 and type of description, such as Fig S1 –[miRNA-mRNA target variation #] – [miRNA cluster].

Fig S1-1-1

**
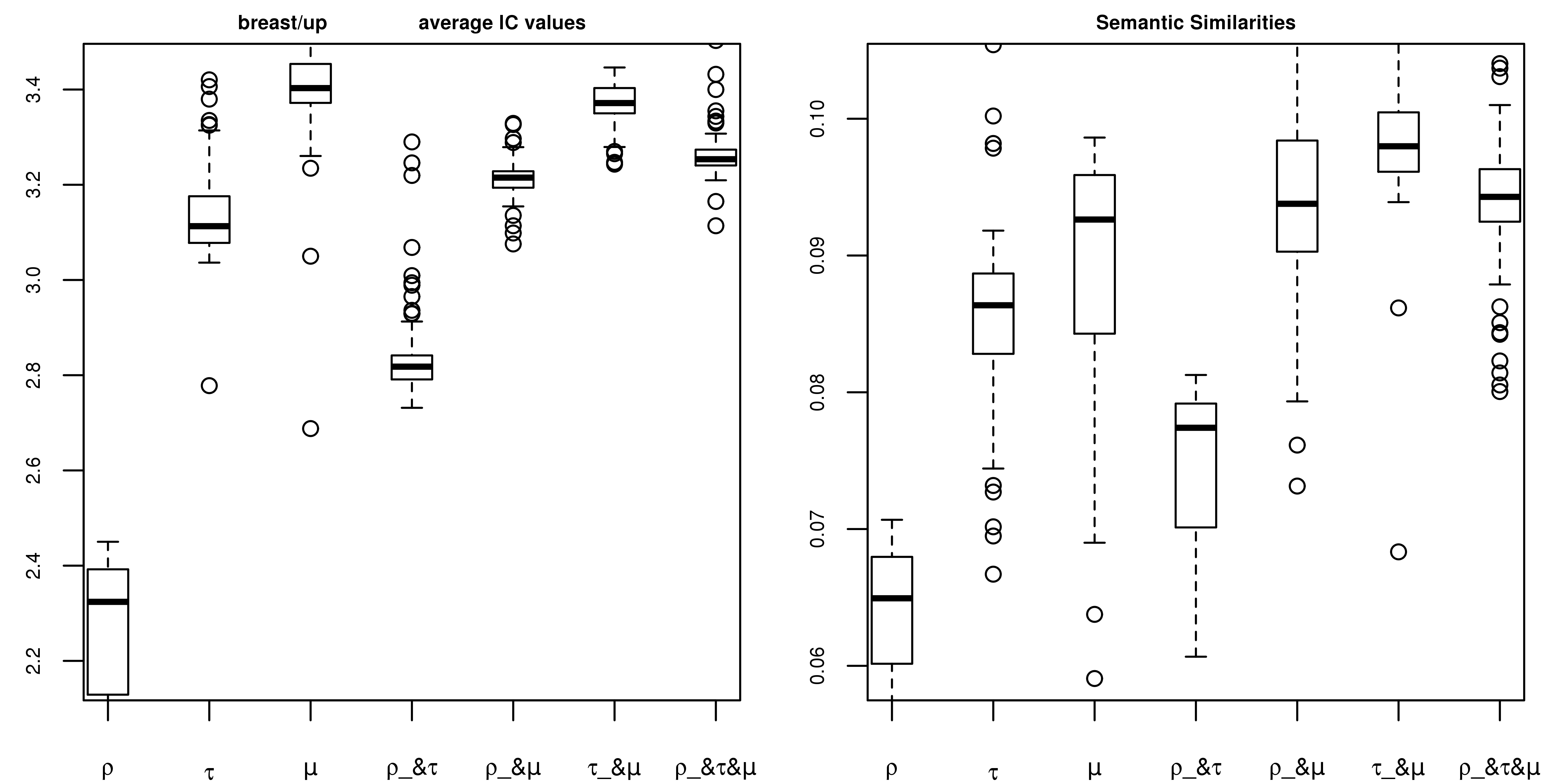
**

Fig S1-1-2


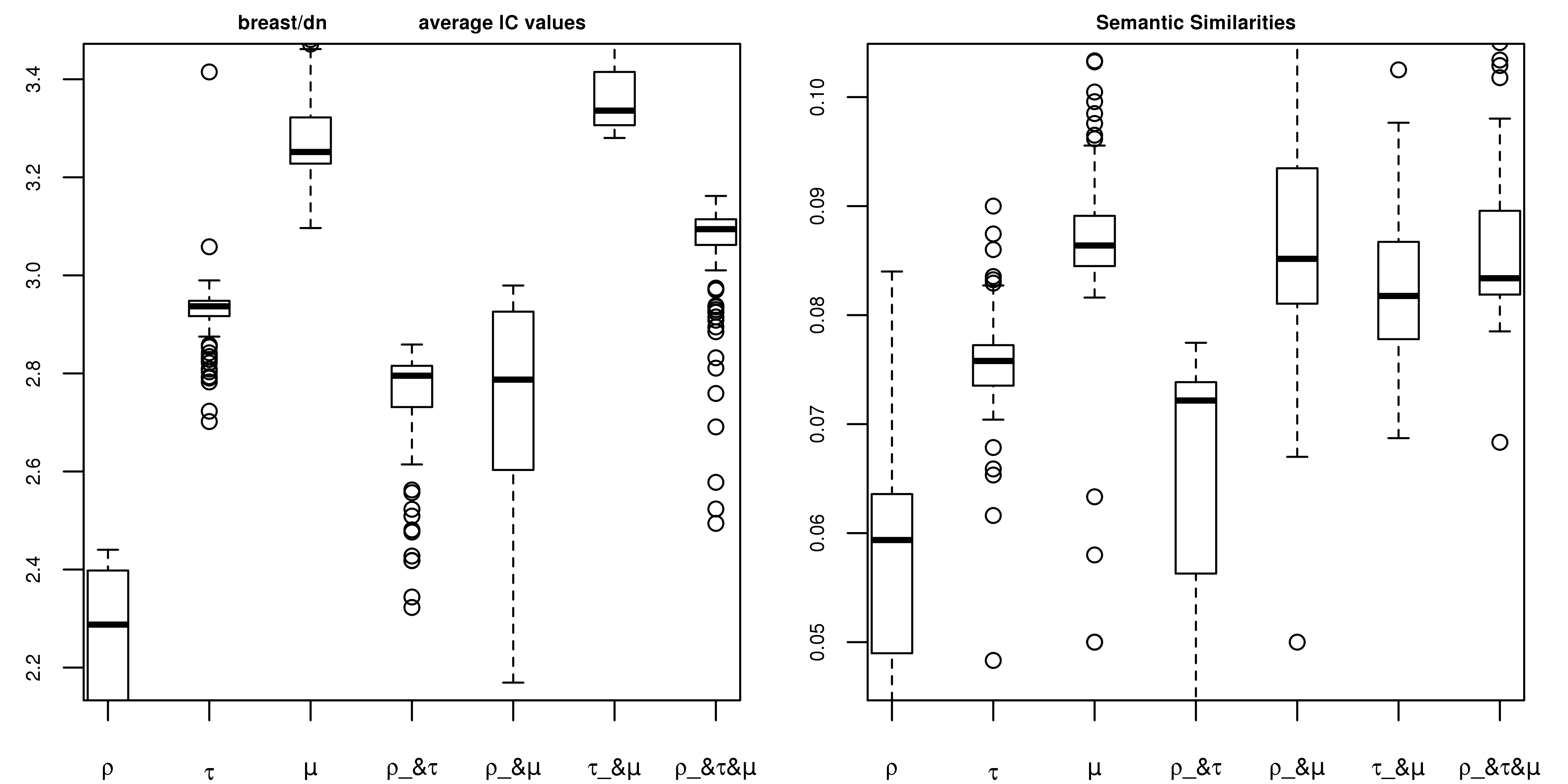


Fig S1-1-3


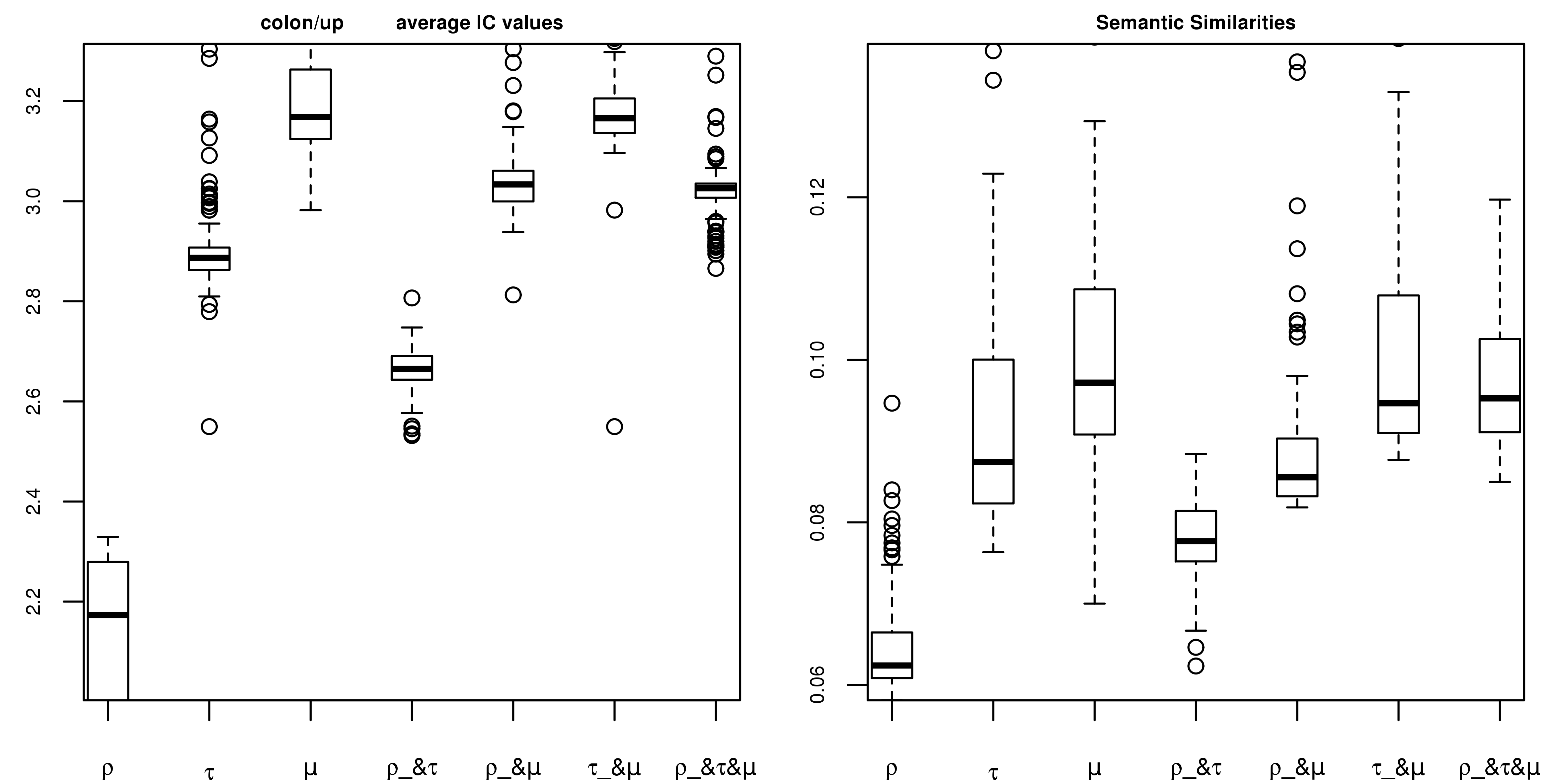


Fig S1-1-5


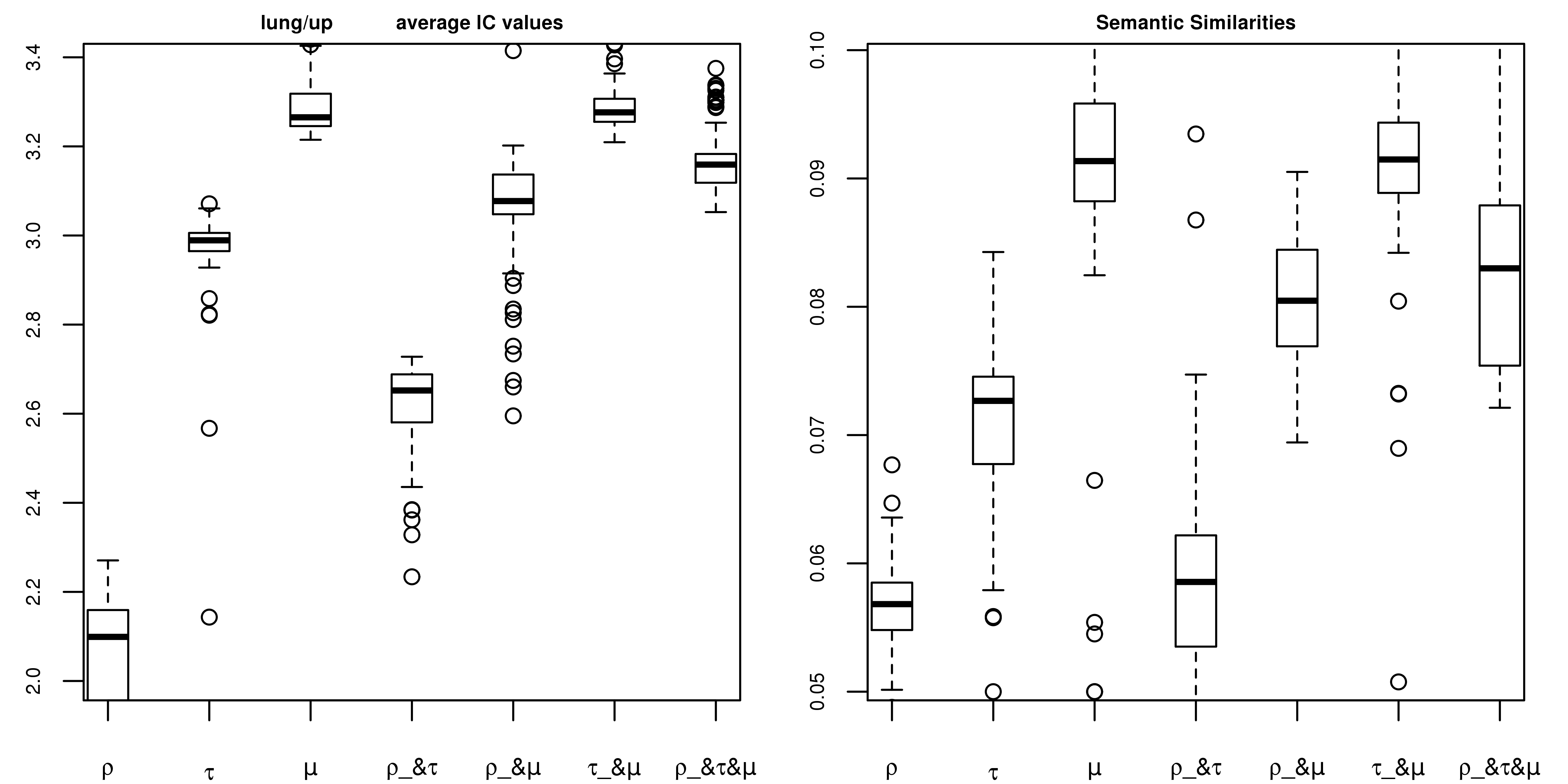


Fig S1-1-6


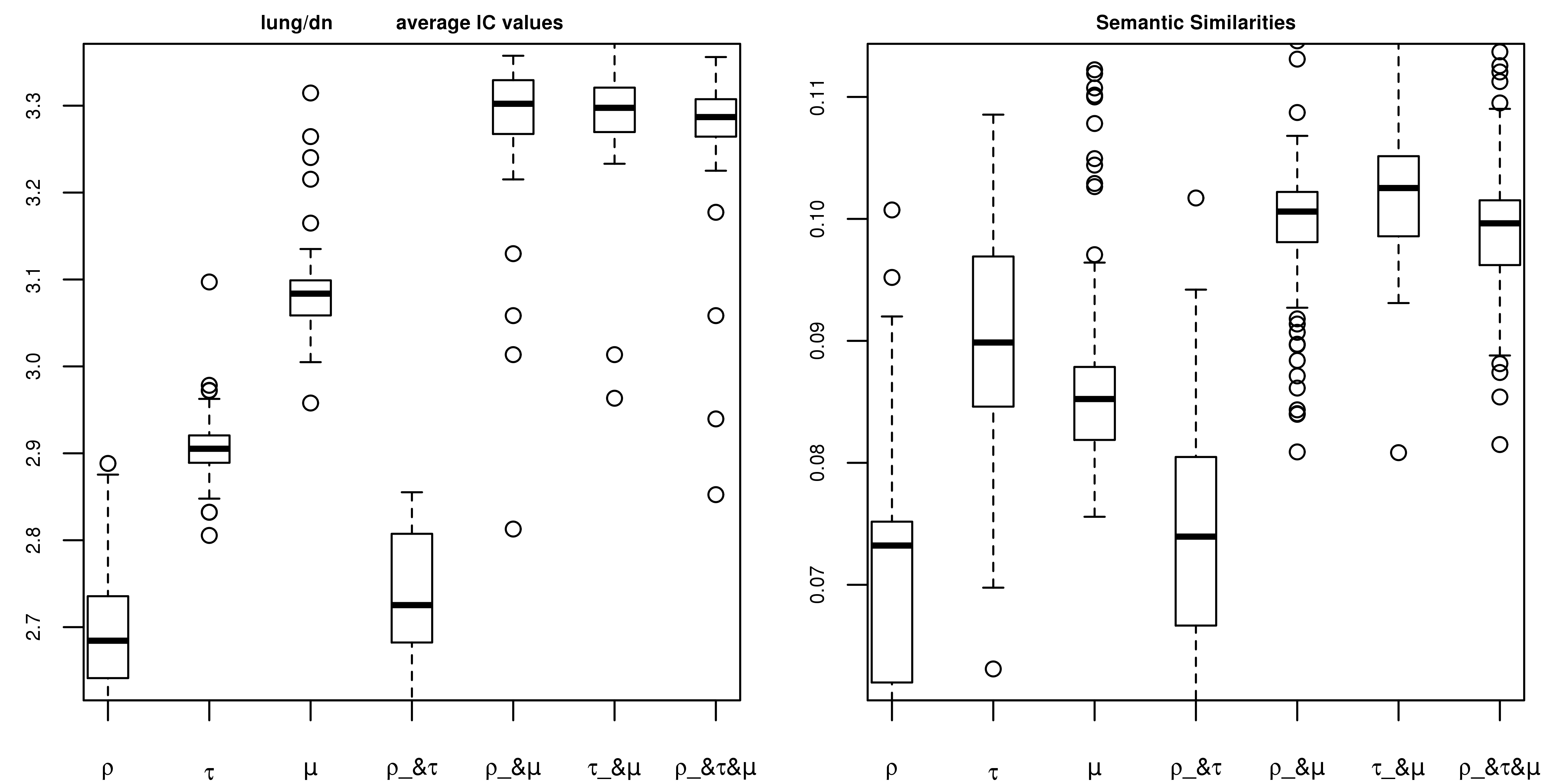


Fig S1-1-7


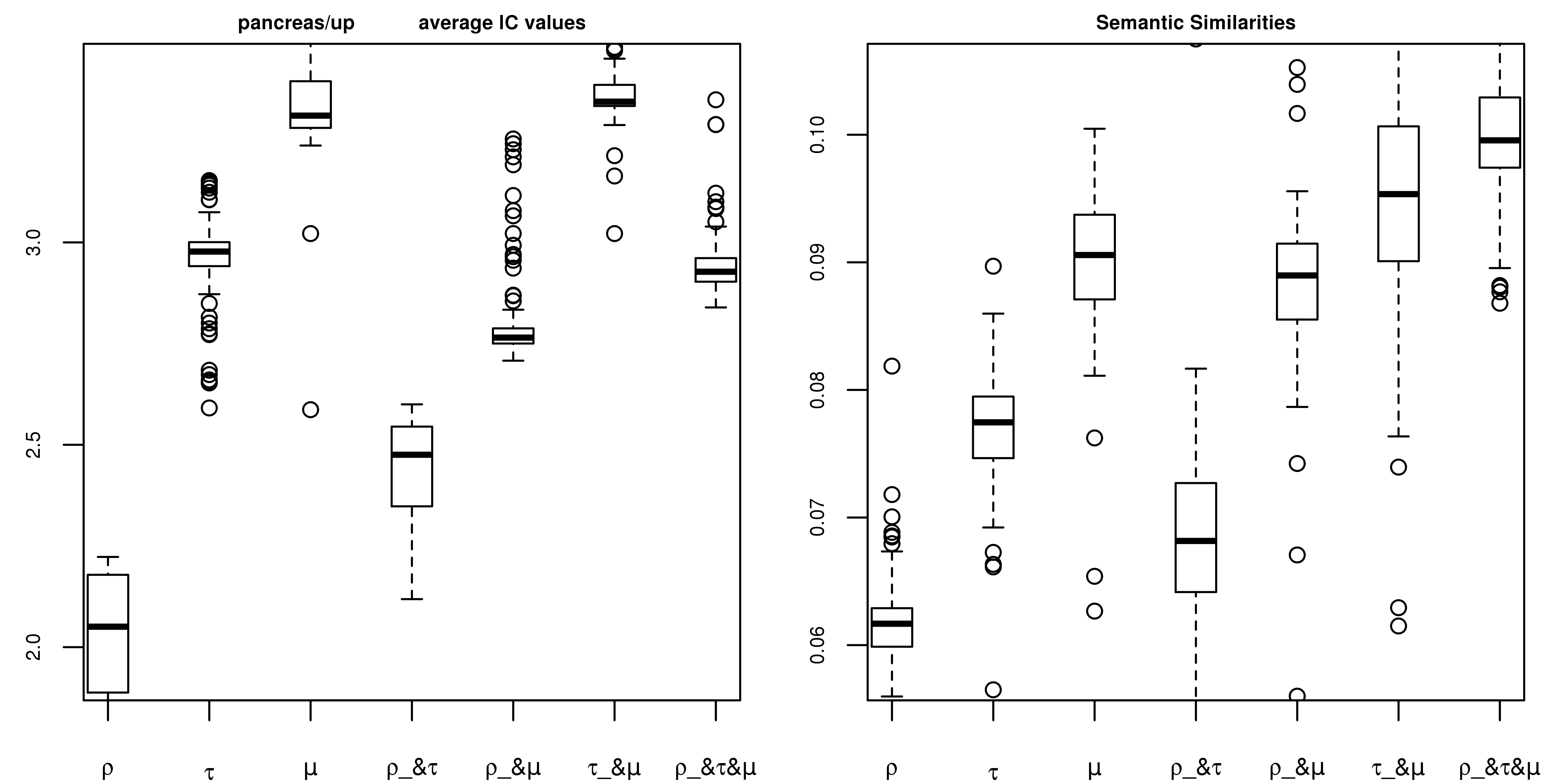


Fig S1-1-8


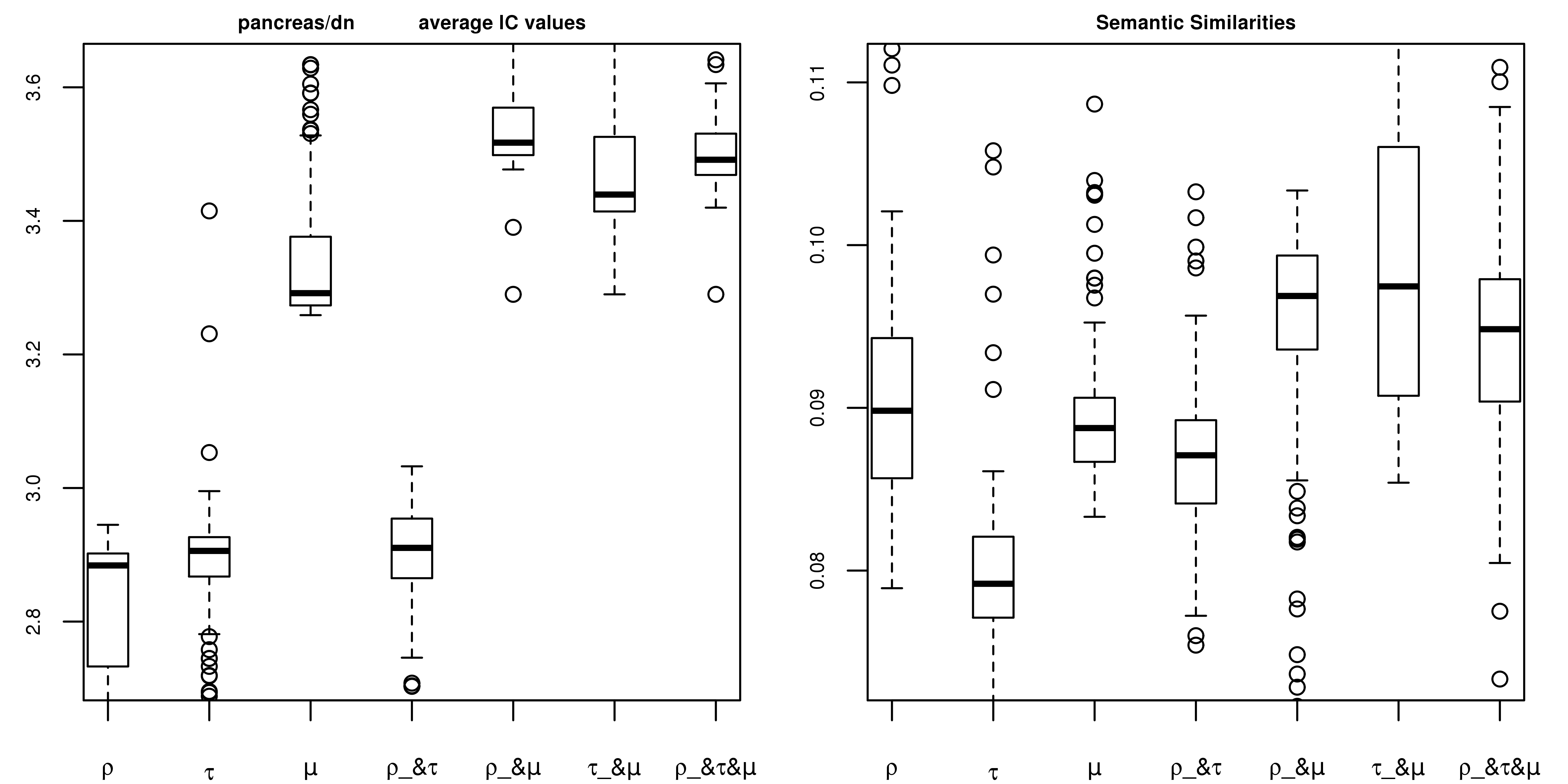


Fig S1-1-9


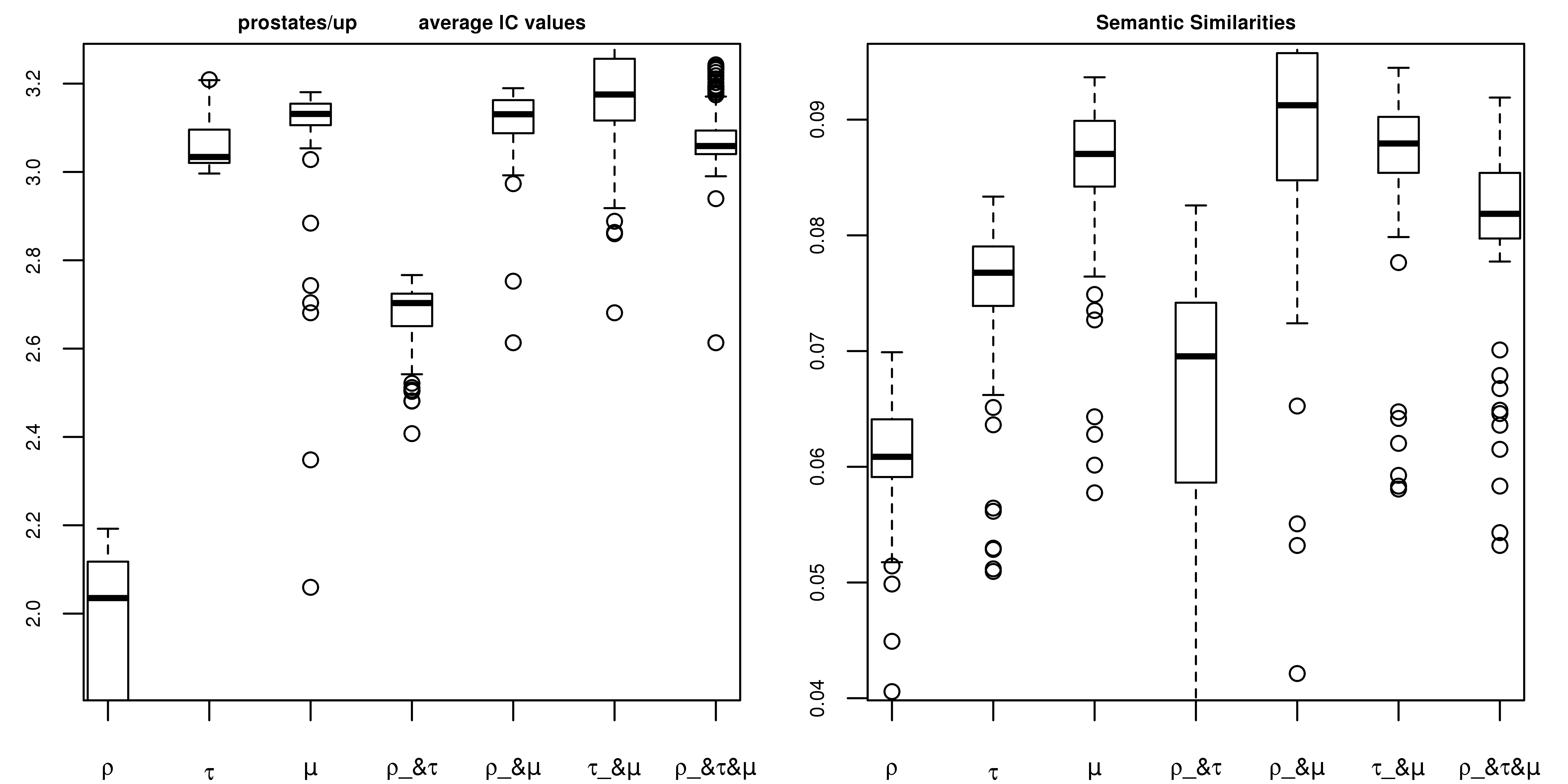


Fig S1-1-10


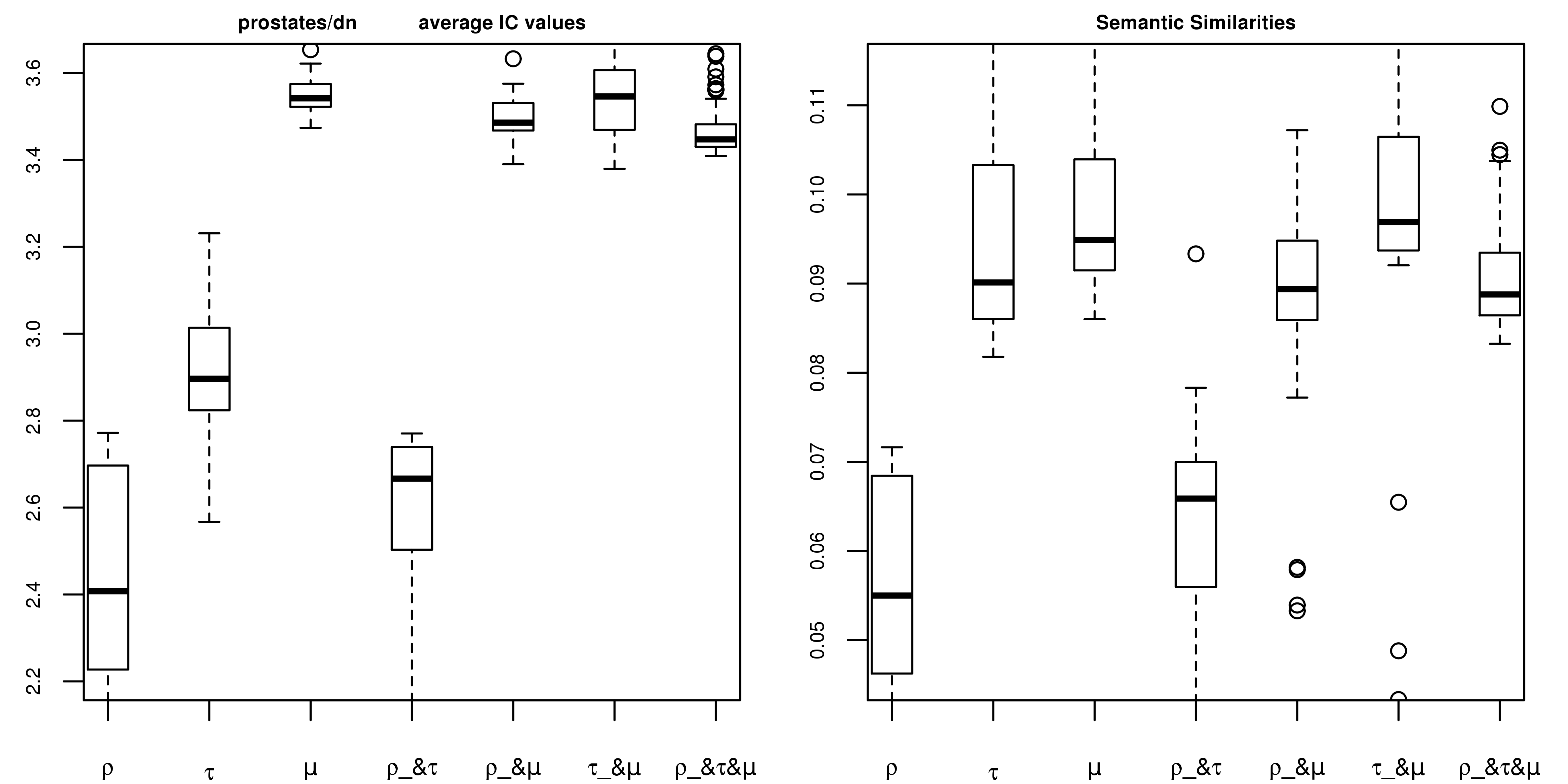


Fig S1-1-11


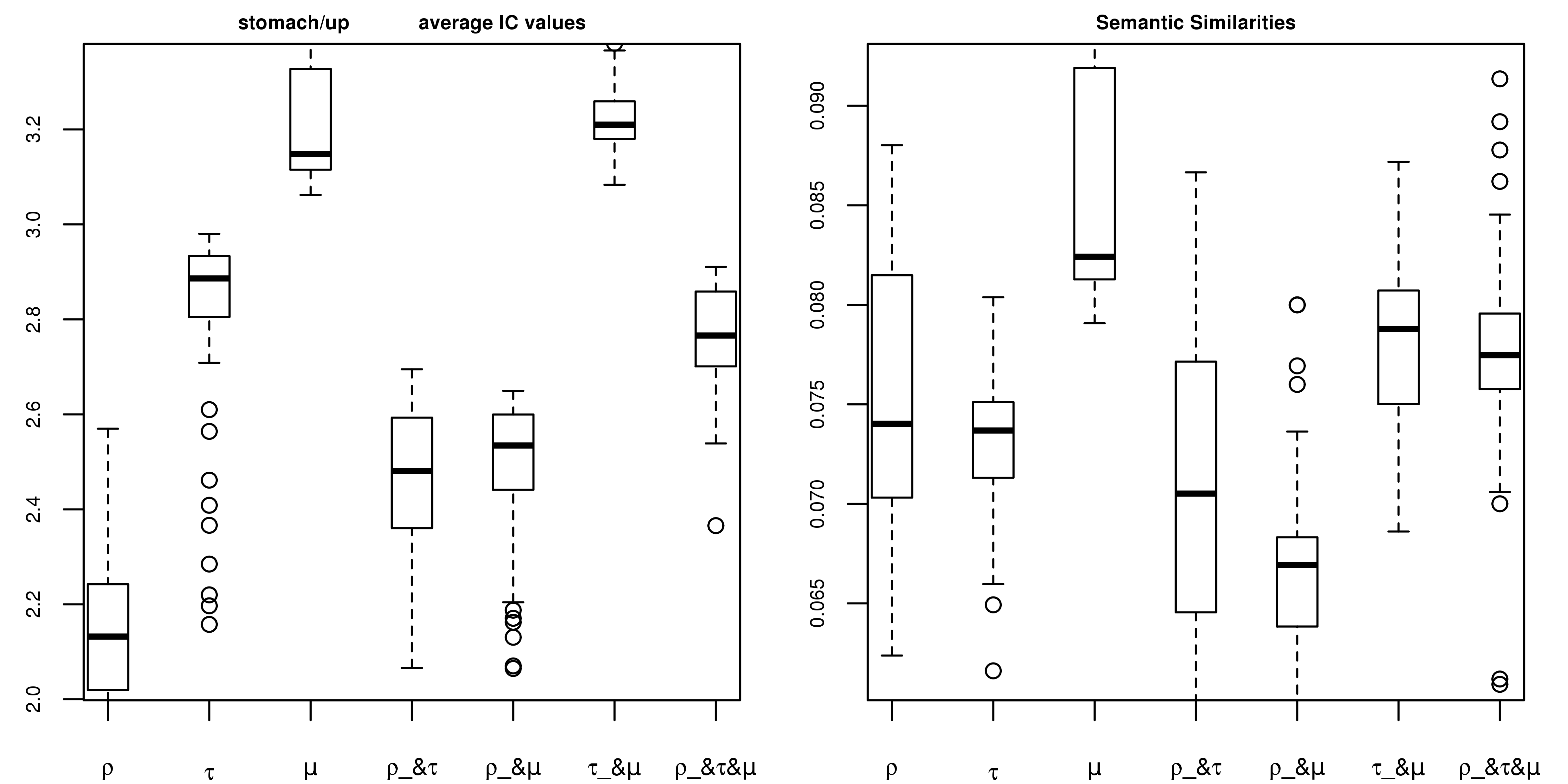


Fig S1-1-12


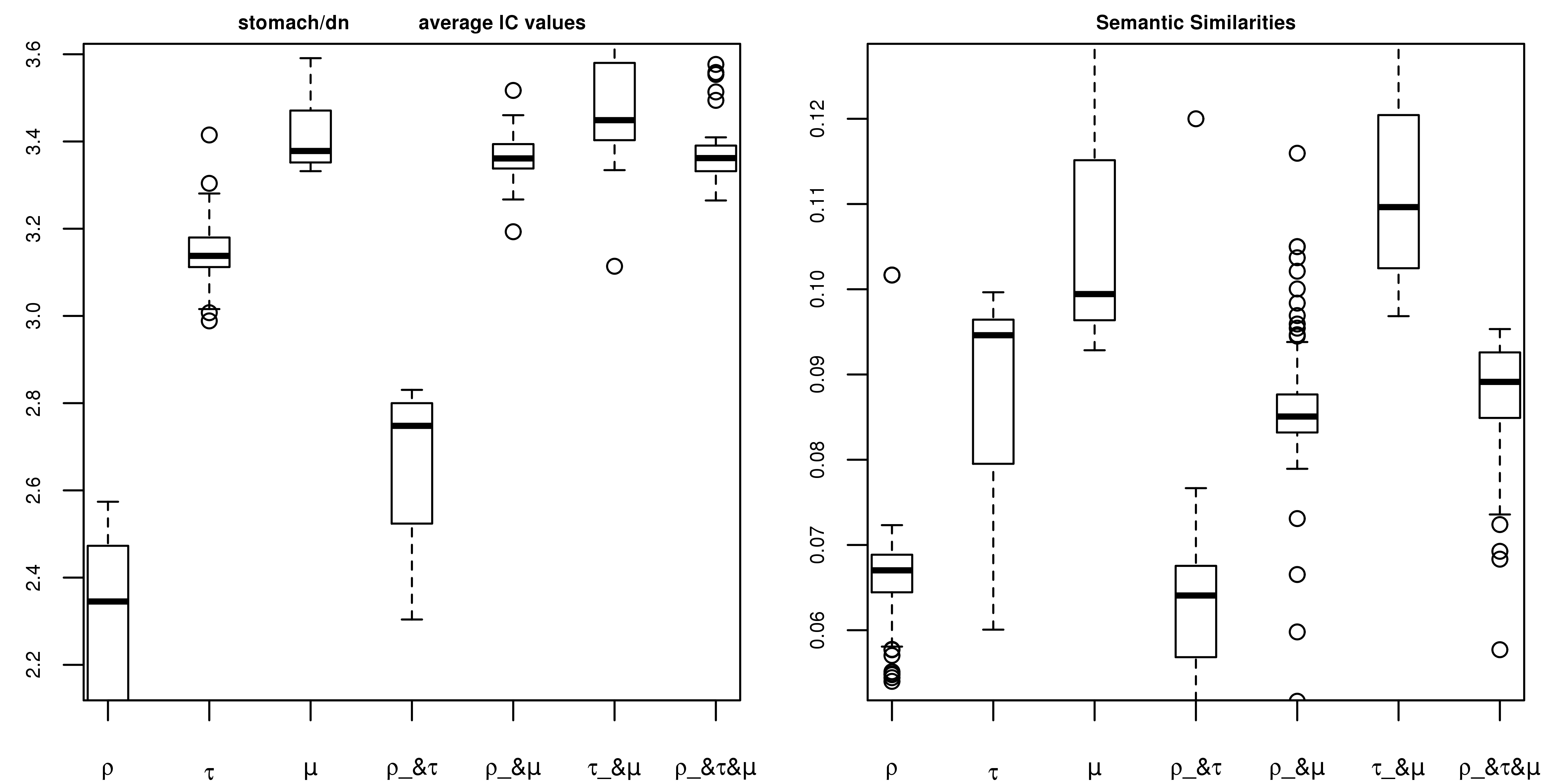


Fig S1-6-1


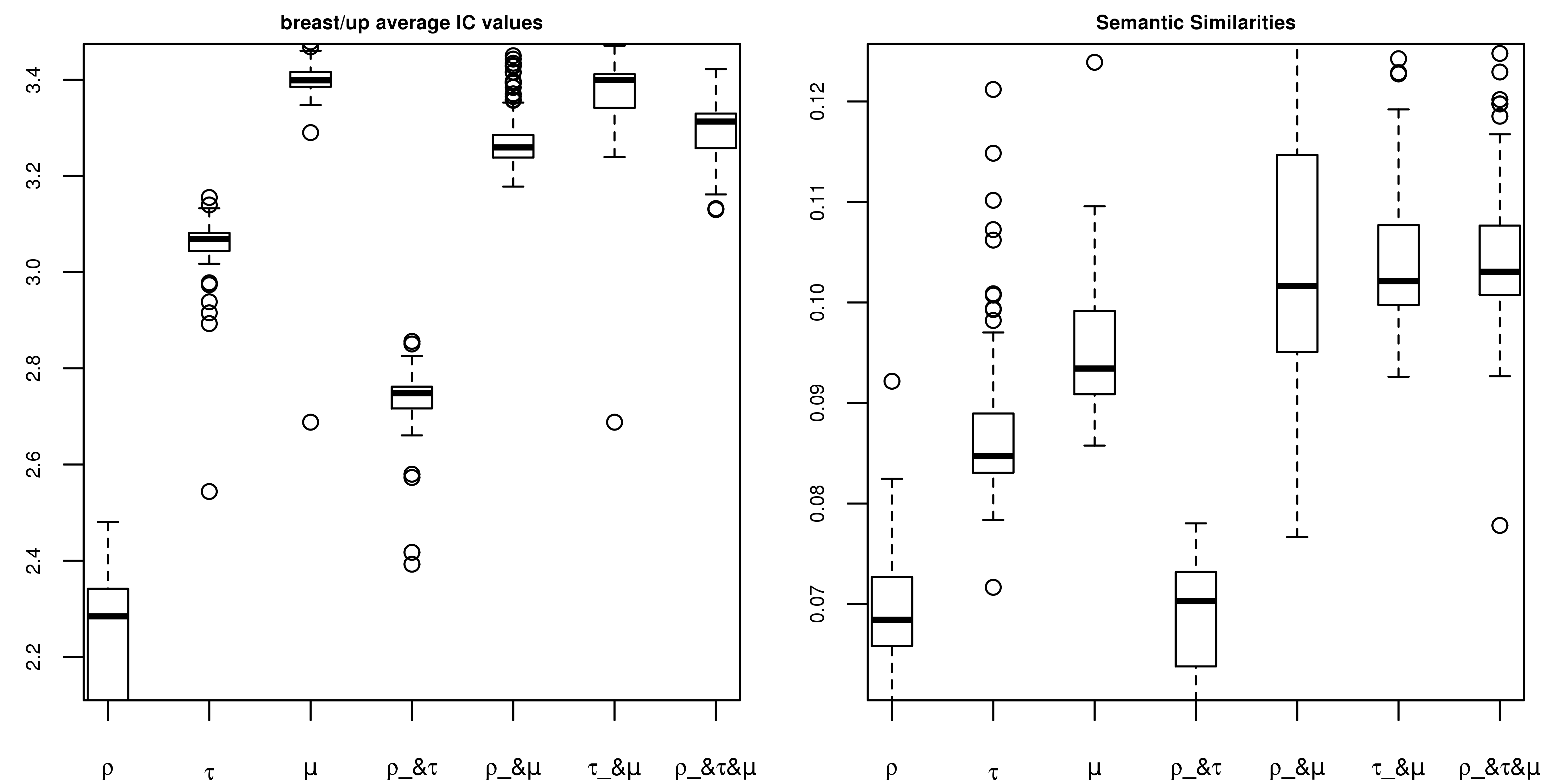


Fig S1-6-2


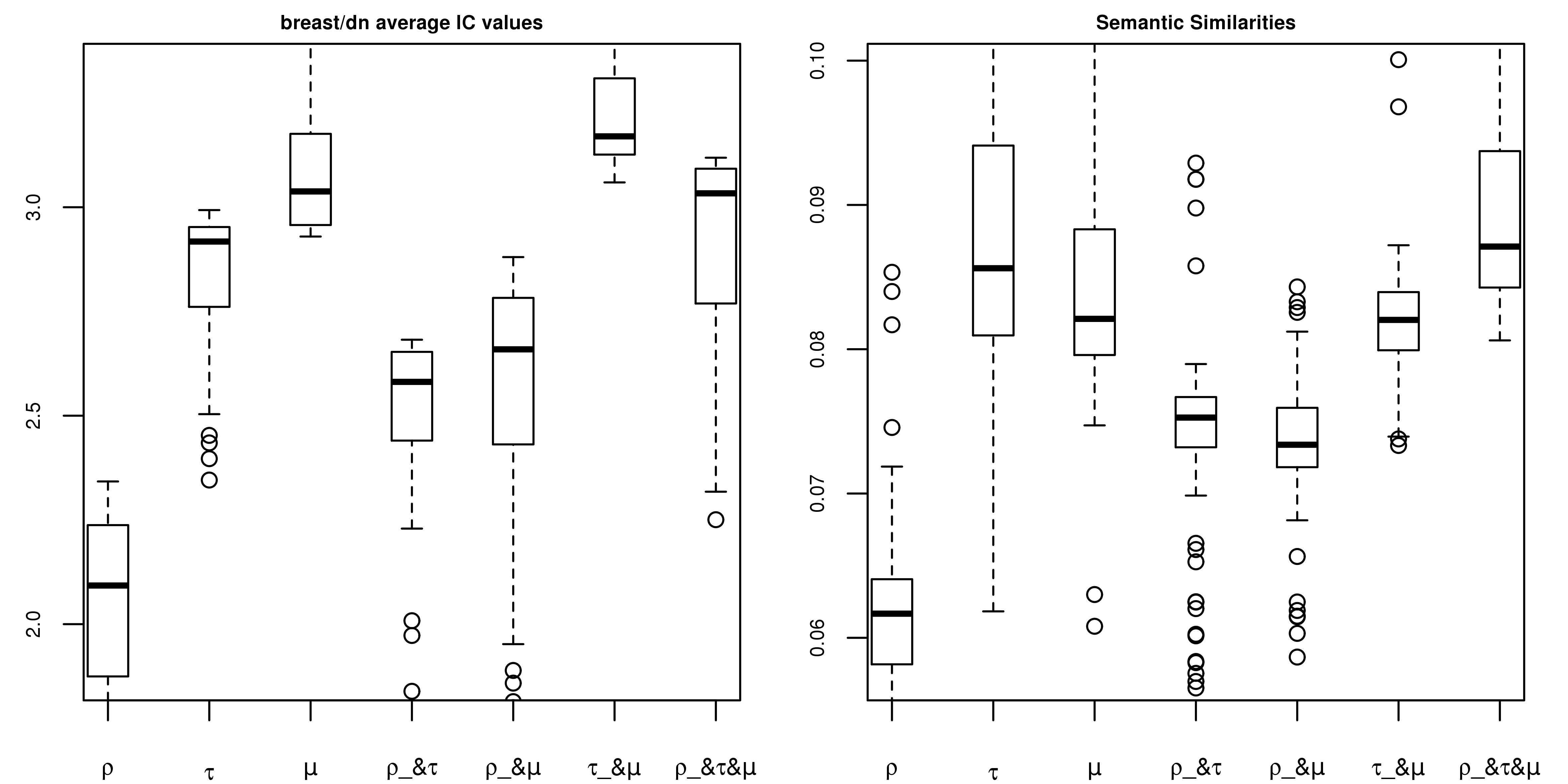


Fig S1-6-3


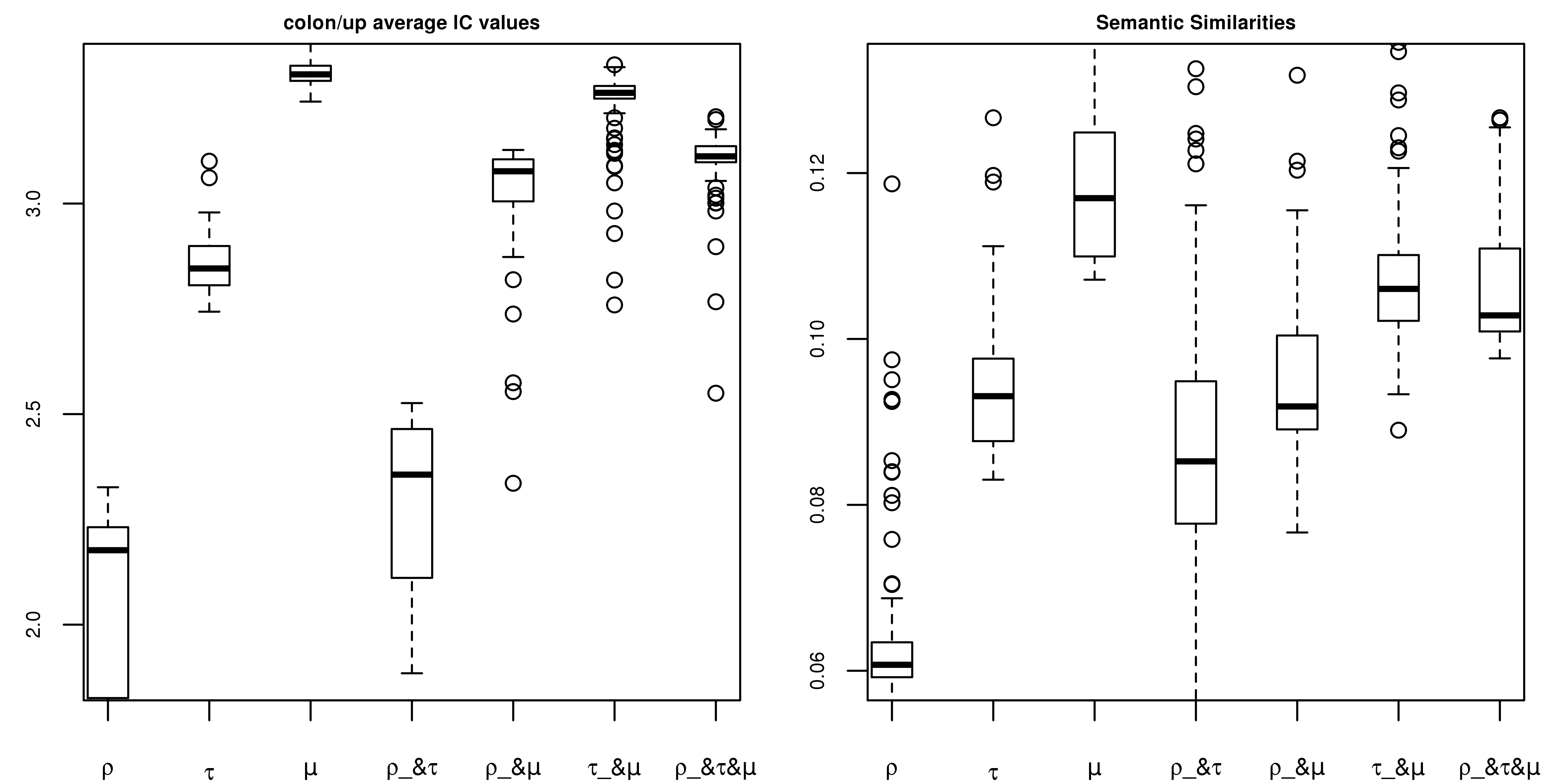


Fig S1-6-5


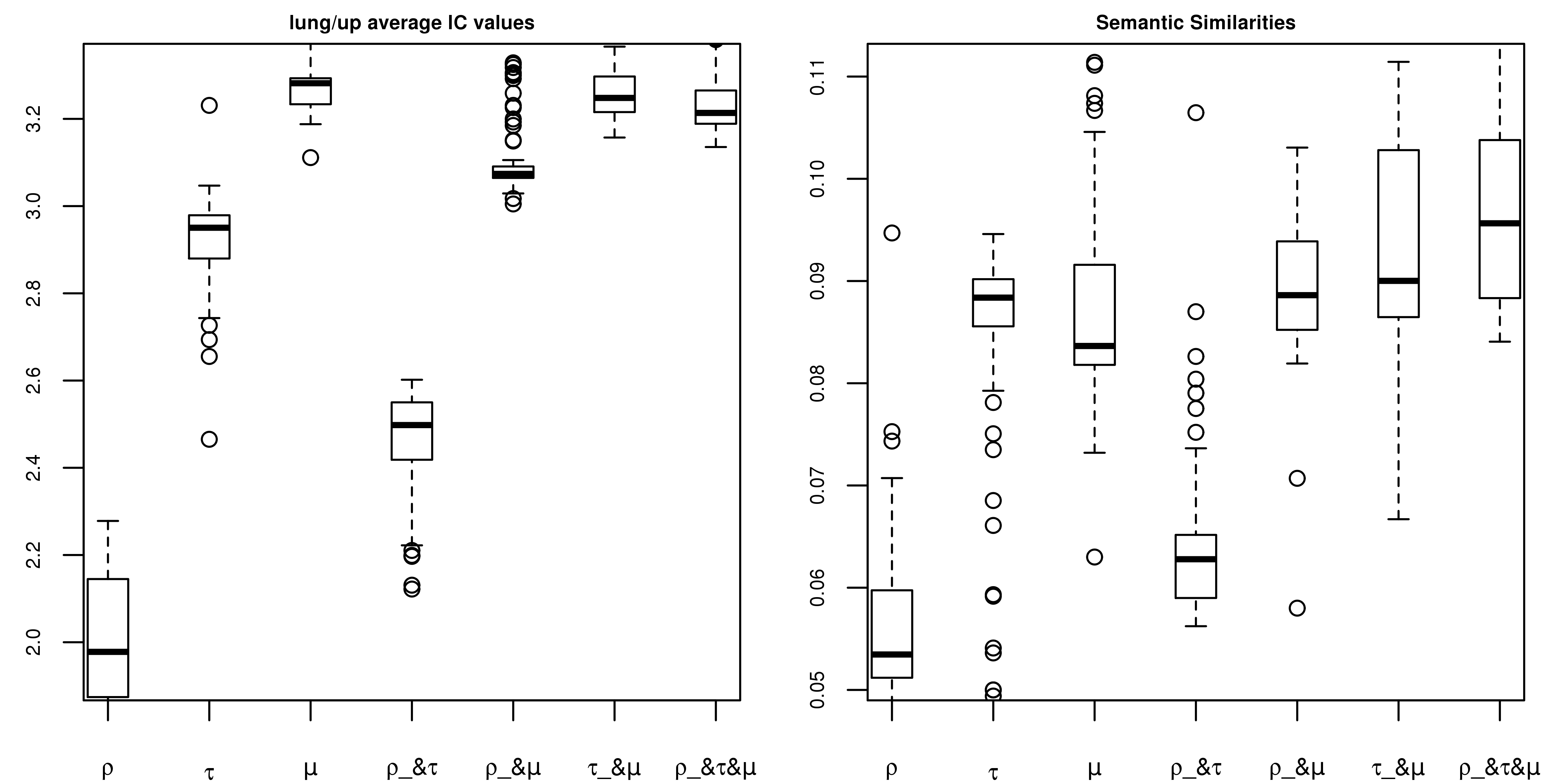


Fig S1-6-6


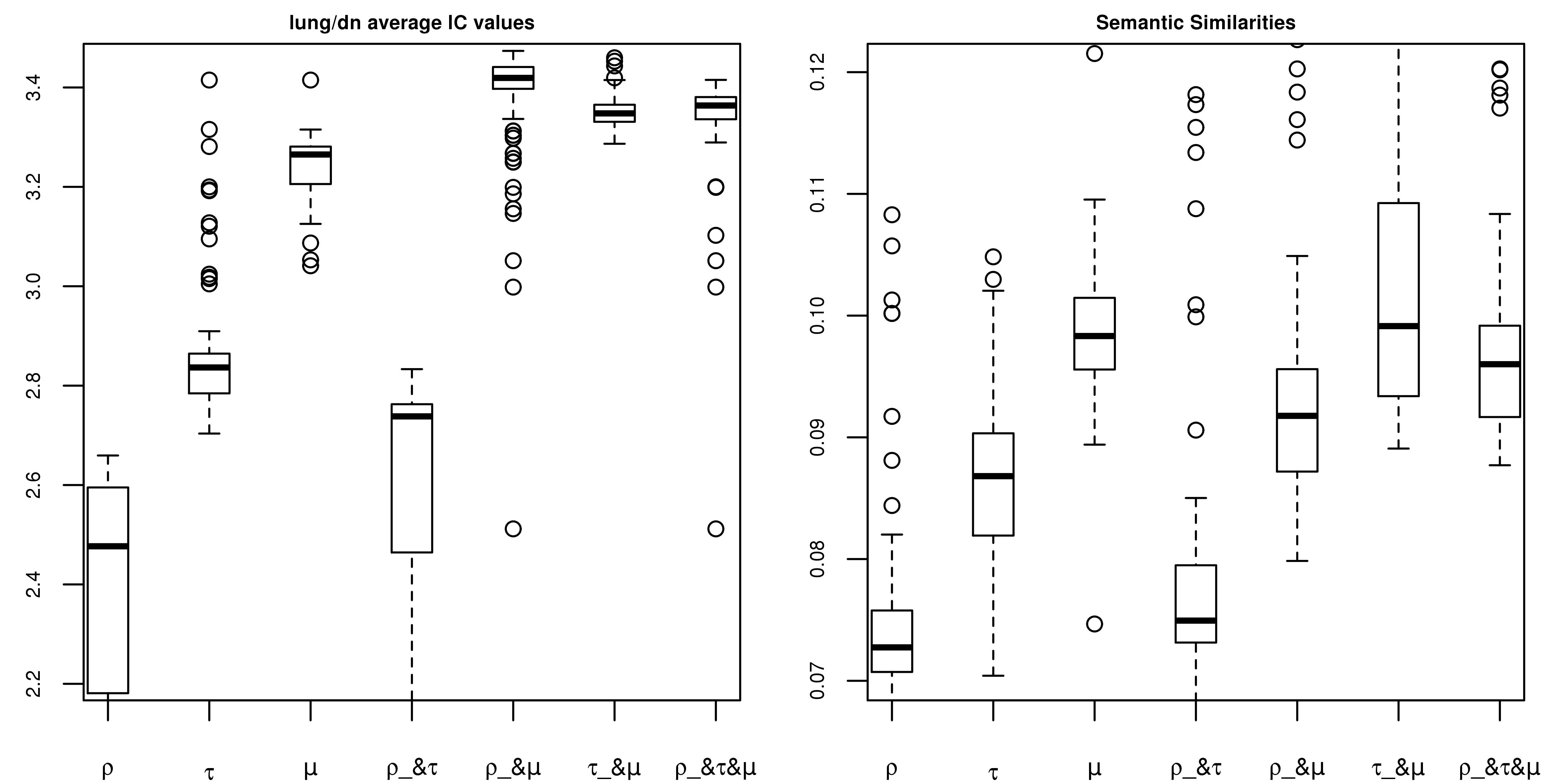


Fig S1-6-7


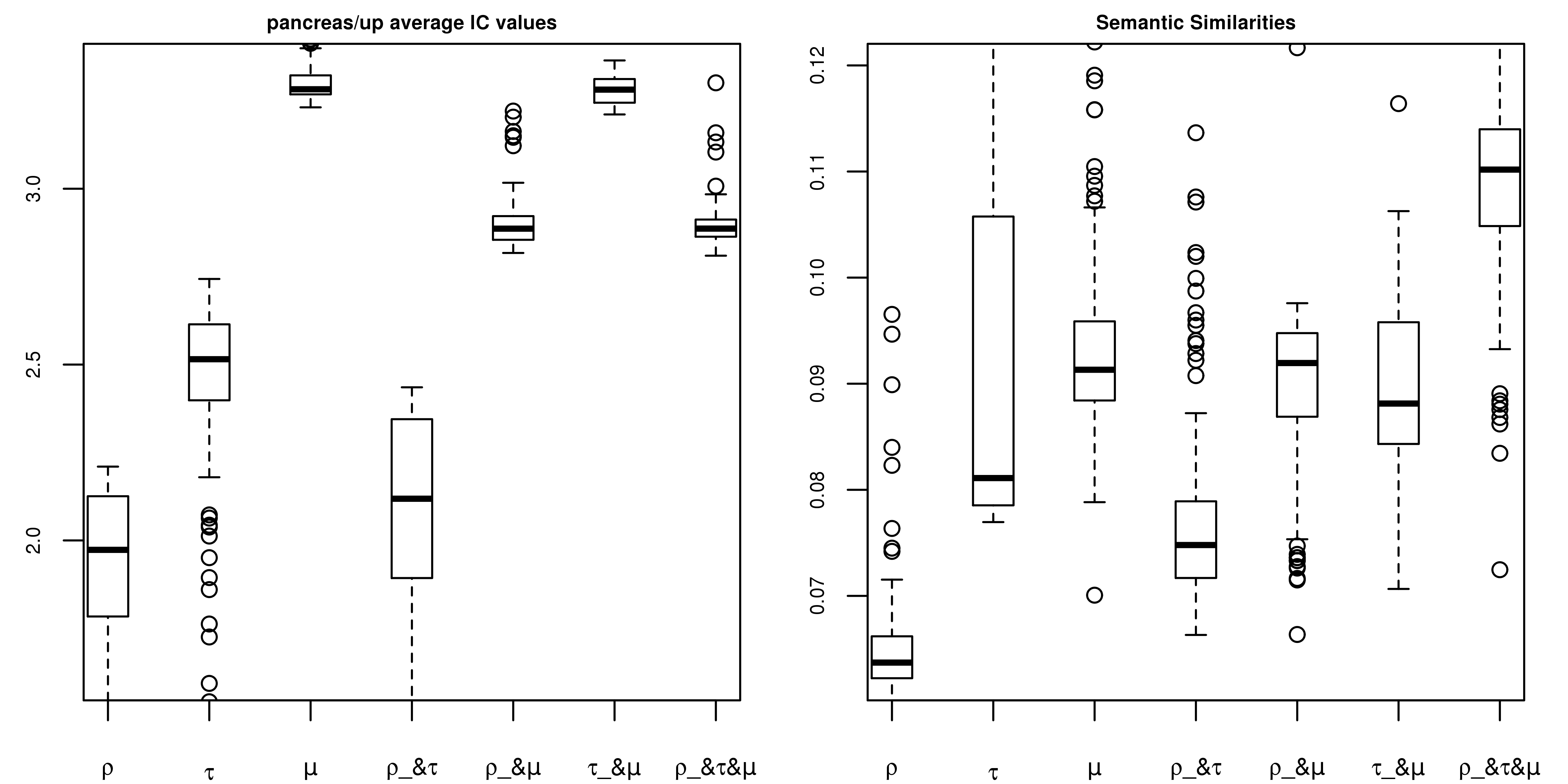


Fig S1-6-8


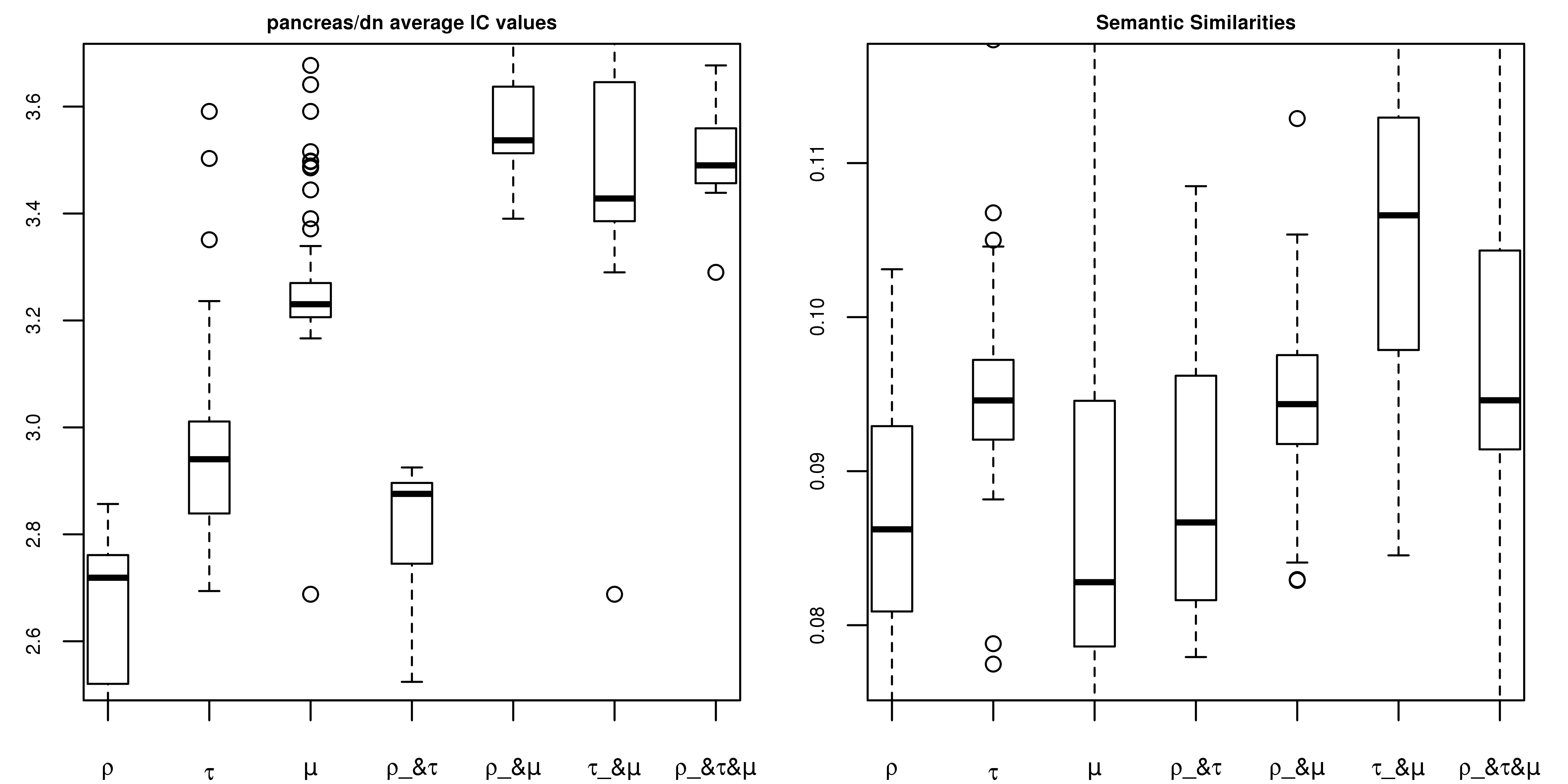


Fig S1-6-9


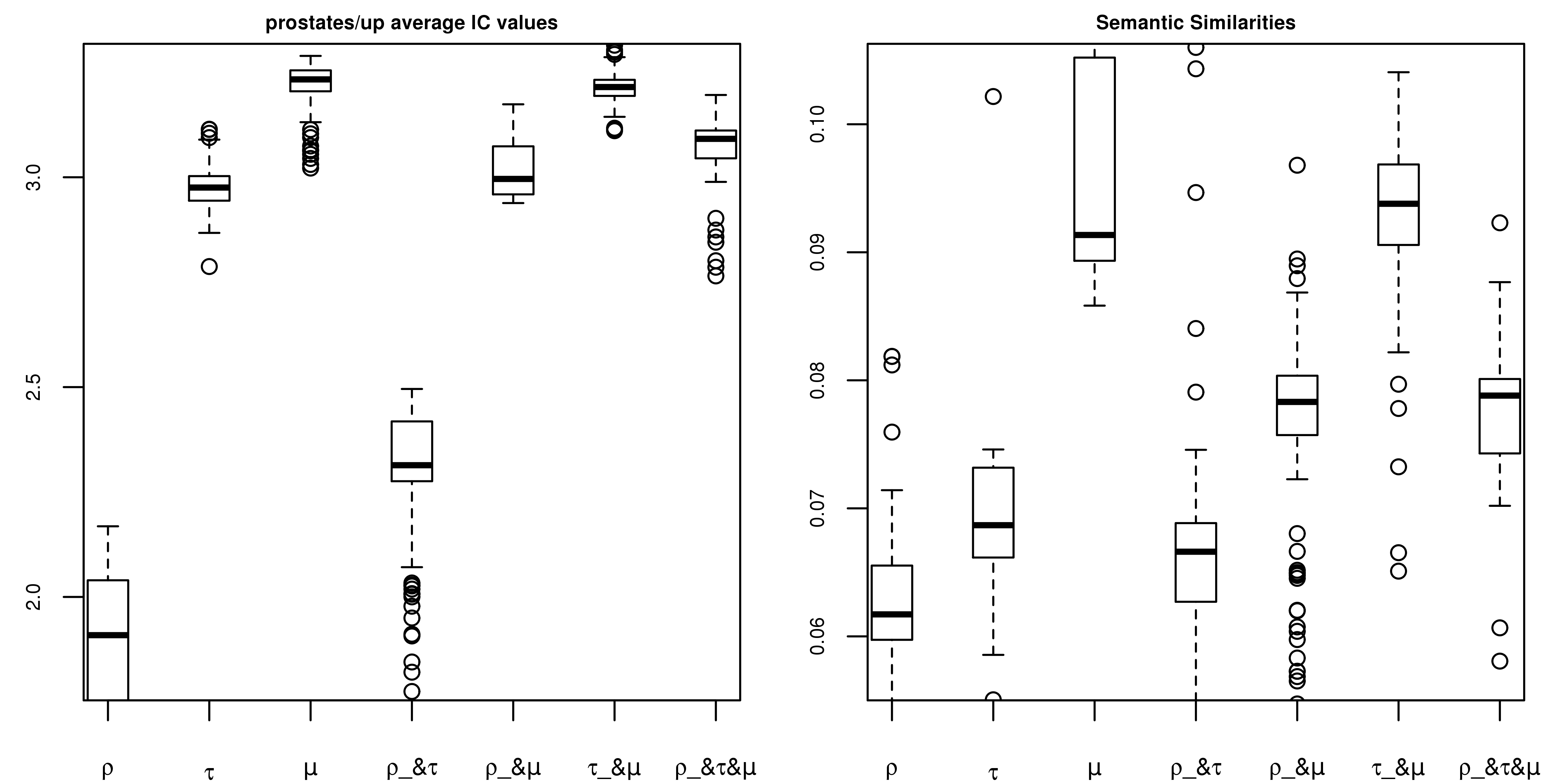


Fig S1-6-10


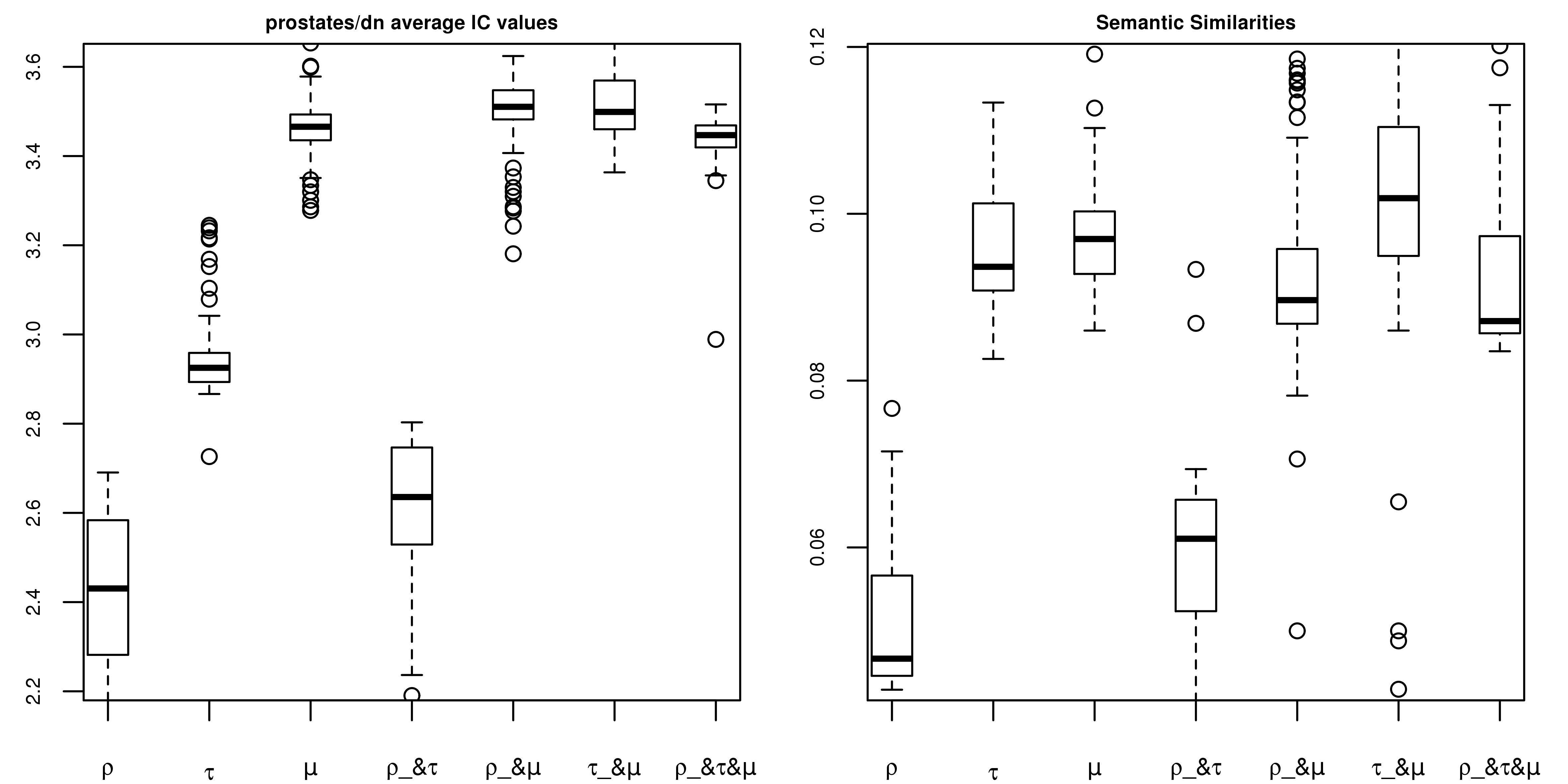


Fig S1-6-11


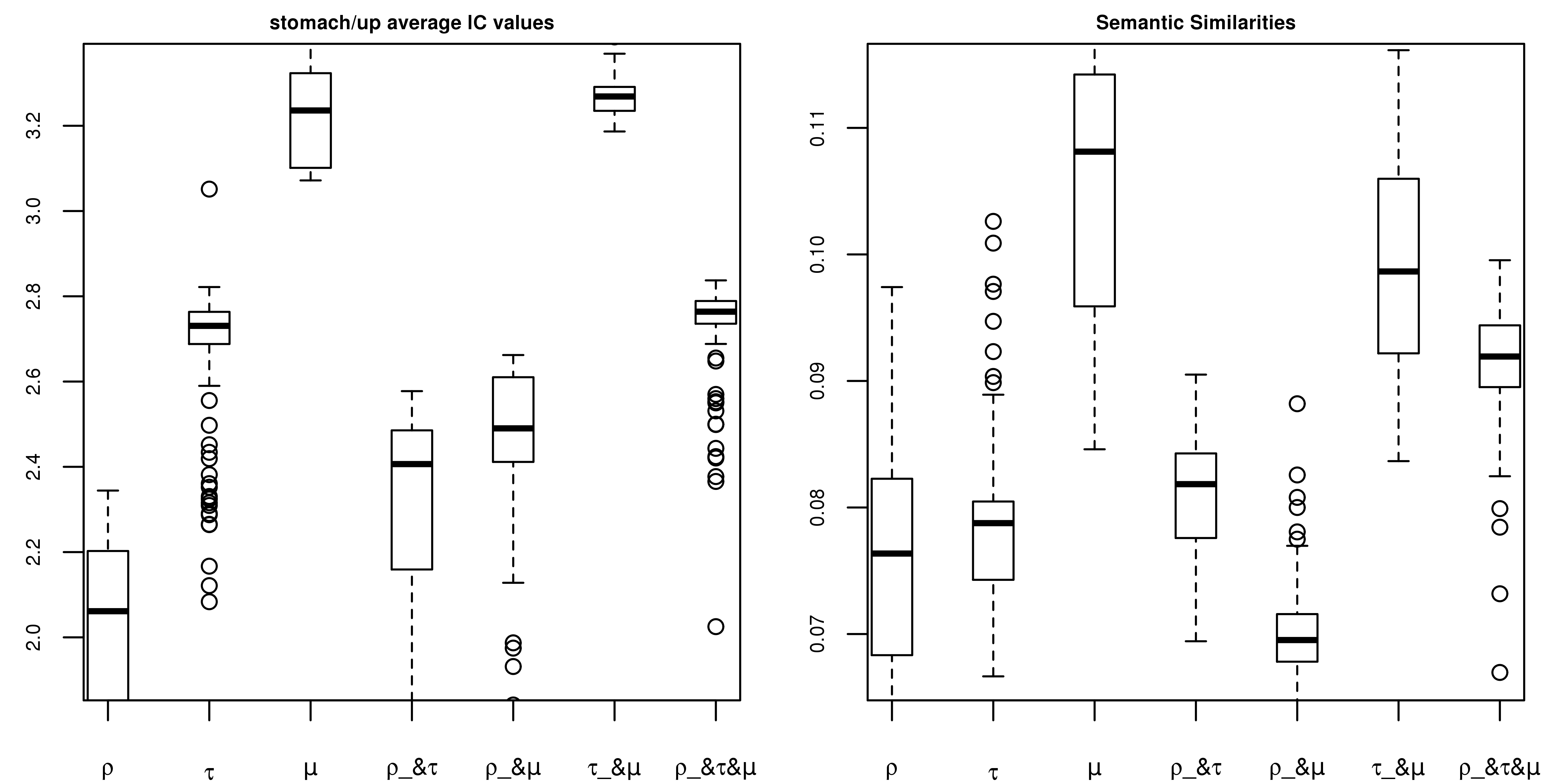


Fig S1-6-12


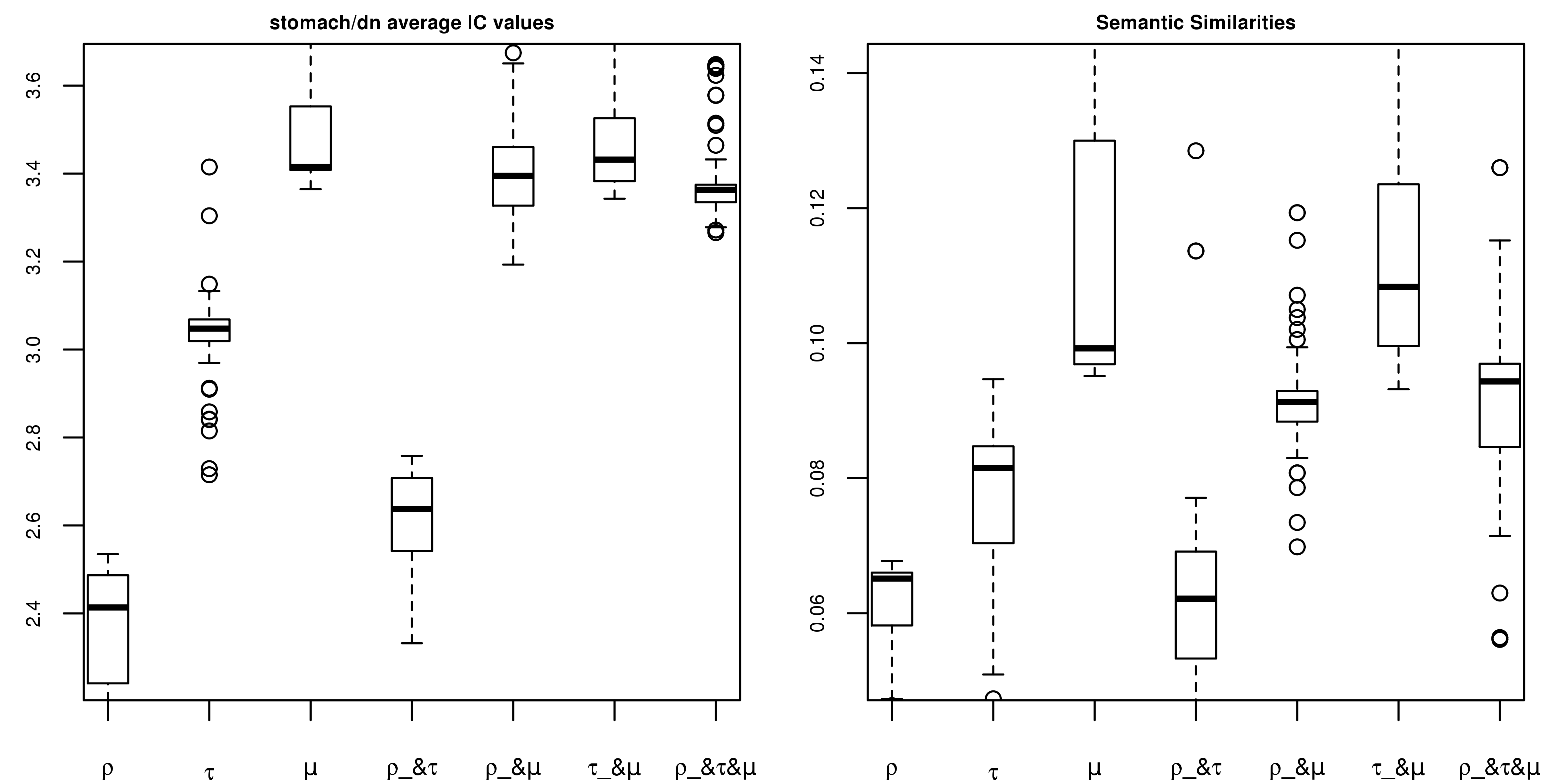

Supplement: Additional file 1 — Supplementary Figures and Tables. This file contains additional figures and tables mentioned in the main text. [file 1471-2164-13-S7-S17-S1.doc]
